# *Stacks*: Building and Genotyping Loci *De Novo* From Short-Read Sequences

**DOI:** 10.1534/g3.111.000240

**Published:** 2011-08-01

**Authors:** Julian M. Catchen, Angel Amores, Paul Hohenlohe, William Cresko, John H. Postlethwait

**Affiliations:** * Center for Ecology and Evolutionary Biology; † Institute of Neuroscience, University of Oregon, Eugene, Oregon 97403

**Keywords:** Illumina, meiotic linkage map, RAD-seq, RAD-tag, zebrafish

## Abstract

Advances in sequencing technology provide special opportunities for genotyping individuals with speed and thrift, but the lack of software to automate the calling of tens of thousands of genotypes over hundreds of individuals has hindered progress. *Stacks* is a software system that uses short-read sequence data to identify and genotype loci in a set of individuals either *de novo* or by comparison to a reference genome. From reduced representation Illumina sequence data, such as RAD-tags, *Stacks* can recover thousands of single nucleotide polymorphism (SNP) markers useful for the genetic analysis of crosses or populations. *Stacks* can generate markers for ultra-dense genetic linkage maps, facilitate the examination of population phylogeography, and help in reference genome assembly. We report here the algorithms implemented in *Stacks* and demonstrate their efficacy by constructing loci from simulated RAD-tags taken from the stickleback reference genome and by recapitulating and improving a genetic map of the zebrafish, *Danio rerio*.

DNA sequencing costs are dropping exponentially (Snyder *et al*. 2010). In addition, short-read sequencing technologies, such as the Illumina HiSeq 2000 that can sequence 100 gigabases of DNA in a few days (http://www.illumina.com/systems/hiseq_2000.ilmn), are expanding experimental space, from fosmids (tens of kilobases), to bacterial artificial chromosomes (hundreds of kilobases), to entire genomes of bacteria (megabases), vertebrates (gigabases), and plants (tens of gigabases). Recent work genotyping 100 stickleback fish at 45,000 loci (Hohenlohe *et al.* 2010) reveals the potential to address questions in population genomics that have not previously been tractable even in model organisms.

Coupling restriction enzyme-based genetic markers, such as RAD-tags (Miller *et al.* 2007), with the Illumina platform (called RAD-seq, Baird *et al.* 2008) allows the rapid and inexpensive construction of genetic linkage maps containing thousands of genetic markers (*e.g.*, 8406 in gar, Amores *et al.* 2011), more than appear on the maps of any but a few intensely investigated species such as mouse (10,000 markers, www.informatics.jax.org/genes.shtml) and economically valuable species such as cow (7063 markers, Arias *et al.* 2009), potato (10,000 markers, van Os *et al.* 2006), and oilseed rape (13,551 markers, Sun *et al.* 2007). Because RAD-seq identifies an enormous number of polymorphisms, single individuals taken directly from the wild possess sufficient genetic diversity to generate high-density, high-quality genetic maps (Amores *et al.* 2011), thus providing genomic information for little-studied species. Exploiting population genomic or genetic mapping datasets with tens of millions of raw reads and millions of genotype calls requires a robust, efficient, and easily useable set of software tools that, unfortunately, have not previously been available.

To solve this problem, we developed *Stacks*, software that identifies loci, either *de novo* or from a reference genome, and calls genotypes using a maximum likelihood statistical model. *Stacks*, named because the restriction enzyme site that anchors each short sequence causes reads at a locus to pile up, is effective for genomic applications ranging from linkage mapping to population genomic and phylogeographic studies.

Here, we report the algorithms implemented in *Stacks*, demonstrate their efficacy through simulation, and test their ability to reconstruct *de novo* a zebrafish genetic map using RAD-tag mapping from the doubled haploid mapping panel (Kelly *et al.* 2000; Postlethwait *et al.* 1994; Shimoda *et al.* 1999; Woods *et al.* 2000; Woods *et al.* 2005). Our results verify the efficacy and efficiency of *Stacks* for inferring genetic loci and automated calling of genotypes.

## Materials and Methods

*Stacks* is implemented by component programs written in C++ and Perl, with the core algorithms parallelized using OpenMP libraries. Table 1 lists *Stacks* components along with a brief description of each. The *Stacks* web interface is implemented in PHP and, along with several component programs, stores and retrieves data from a MySQL database. The web interface interacts with the database using the MDB2 Pear module. *Stacks* is available as open source software under the GPL license and can be downloaded from http://creskolab.uoregon.edu/stacks/.

**Table 1:** *Stacks* component programs

Program	Description	Inputs	Database interaction
process_radtags.pl	Cleans raw Illumina reads, outputs FASTA/FASTQ files.	Raw Illumina reads	No
ustacks (unique stacks)	Builds loci *de novo* and detects haplotypes in one individual.	Cleaned FASTA/FASTQ files	No
cstacks (catalog stacks)	Merges loci from multiple individuals to form a catalog.	ustacks, tab-separated files	No
sstacks (search stacks)	Matches loci from an individual against a catalog.	ustacks and cstacks, tab-separated files	No
markers.pl	Calls mappable markers from parental loci.	None	Yes
index_radtags.pl	Indexes the database for use by the web interface.	None	Yes
denovo_map.pl	Executes ustacks on each individual, builds a catalog with cstacks, and matches individuals against the catalog with sstacks. Calls markers with markers.pl and indexes the database with index_radtags.pl.	Cleaned FASTA/FASTQ files	Yes
genotypes.pl	Calls genotypes in a map cross population and outputs markers for use by JoinMap or r/QTL.	None	Yes
pstacks (population stacks)	Takes cleaned reads aligned to a reference genome, builds stacks based on the genomic locations of the reads, and detects haplotypes in one individual.	Bowtie or SAM sequence alignments	No
ref_map.pl	Executes pstacks on each individual, builds a catalog with cstacks, and matches individuals against the catalog with sstacks. Calls markers with markers.pl and indexes the database with index_radtags.pl.	Cleaned FASTA/FASTQ files	Yes
sort_read_pairs.pl	Given a set of *Stacks* data and a set of cleaned, paired-end Illumina reads, outputs one FASTA file for each stack consisting of the paired-end reads associated with reads in that stack.	ustacks output files, cleaned FASTA/FASTQ files	No
load_sequences.pl	Loads a set of loci-associated sequences (*e.g.*, RNA-seq ESTs) into the database.	FASTA file containing sequences	Yes
export_catalog.pl	Exports sequences from the database, including loci and loci-related sequences.	None	Yes

### Simulating RAD-tags to test performance

The *Stacks* core component program is ustacks, which identifies unique loci *de novo*. To test ustacks, we created simulated datasets from the stickleback reference genome (BROAD S1, Ensembl version 59) by extracting 45,547 reads each 60 bp long in both directions at each SbfI restriction enzyme cut site (CCTGCA^v^GG) (Figure S1A). We re-diplodized the genome *in silico* by creating alleles (Figure S1B) into which we uniformly introduced single nucleotide polymorphisms (SNP) at a rate of 0.5%. We “sequenced” each allele to a depth determined by a draw from a Poisson distribution at three different mean sequencing depths (10×, 20×, and 40×) (Figure S1C). For each “sequenced” read, we simulated sequencing errors at a rate that increased linearly along the sequence to mimic Illumina reads (Figure S1D). We investigated three mean error rates (0.5%, 1%, and 3%) to cover normal to high error rates. Each simulation run involved 10 replicates. For each dataset, ustacks was executed setting the *within-individual distance* parameter to two nucleotides and the *stack-depth* parameter to three identical reads.

### Constructing a dense zebrafish map

DNAs from the gynogenetic doubled haploid zebrafish HS mapping panel (Kelly *et al.* 2000; Woods *et al.* 2005) were prepared for RAD-tags according to Amores *et al.* (2011) and Etter *et al.* (2011). Progeny were sequenced with 60 bp reads in three Illumina GAII lanes, resulting in 70,921,725 raw reads, of which 57,451,403 were retained after cleaning. Because DNA of the original female parent was no longer available, we combined all reads from her gynogenetic progeny to create a synthetic maternal genome and processed the content through *Stacks*. We executed the *Stacks* pipeline with a *stack-depth* parameter of three and a *within-individual distance* parameter of two and constructed a linkage map using JoinMap (Van Ooijen 2006). While *Stacks* has no limit to the number of markers it can handle, JoinMap is limited to about 8000 markers. To work around this deficiency in JoinMap, we subdivided *Stacks* output into overlapping datasets small enough for JoinMap to handle and then ran JoinMap to construct linkage groups, using markers shared in overlapping datasets to identify corresponding linkage groups. Linkage group–specific datasets with fewer than 8000 markers each were finally loaded into JoinMap to identify locus order.

Besides comparing the RAD-tag map to a previously published meiotic map, we also aligned RAD-tag markers to the physical genome (Zv9, Ensembl version 61) by BLASTn. These searches used an e-value cutoff of 1 × 10^−17^ (to allow for sequencing errors and for polymorphisms between the reference genome and the HS panel) and required a unique best hit to the reference genome or a top hit with a raw BLAST score at least an order of magnitude greater than the second best hit with 70% of the query sequence aligned. Genotypes for markers present in at least 36 of the 42 HS map cross individuals were exported into JoinMap 4.0 (Van Ooijen 2006). Linkage between markers, recombination rate, and map distances were calculated using the Kosambi mapping function and the maximum likelihood function in JoinMap. Markers were grouped at an initial logarithm of the odds (LOD) threshold of 7.0, and small linkage groups were incorporated using the strong cross-link feature of JoinMap at a minimum LOD of 5.0. Markers with strong segregation distortion or that appeared unlinked at LOD < 5.0 were excluded.

## Results

We designed *Stacks* as a modular pipeline to efficiently curate and assemble large numbers of short-read sequences from multiple samples. *Stacks* identifies loci in a set of individuals, either *de novo* or aligned to a reference genome, and then genotypes each locus. *Stacks* incorporates a maximum likelihood statistical model to identify sequence polymorphisms and distinguish them from sequencing errors. *Stacks* employs a Catalog to record all loci identified in a population and matches individuals to that Catalog to determine which haplotype alleles are present at every locus in each individual. *Stacks* stores results in a MySQL database and displays them through a web interface that facilitates marker annotation. The database also allows linking markers to other sequence information, such as RNA-seq data (Mortazavi *et al.* 2008). *Stacks* can export data as genotypes for JoinMap (Van Ooijen 2006) or R/qtl (Broman *et al.* 2003) or as a set of observed haplotypes for a general population.

Because *Stacks* was originally designed to build meiotic maps (Amores *et al.* 2011), some pipeline terminology pertains to genetic mapping, but *Stacks* can be used for nearly any analysis using genomically localized short-read sequences. We describe here how the pipeline functions to build a genetic map *de novo*, and then how it can use a reference genome. Finally, we describe the testing of *Stacks* by simulation and by reconstructing a zebrafish genetic map. The *Stacks* component programs are discussed below and described in Table 1.

### Building markers for a genetic map *de novo*

#### Overview:

**Figure 1:**
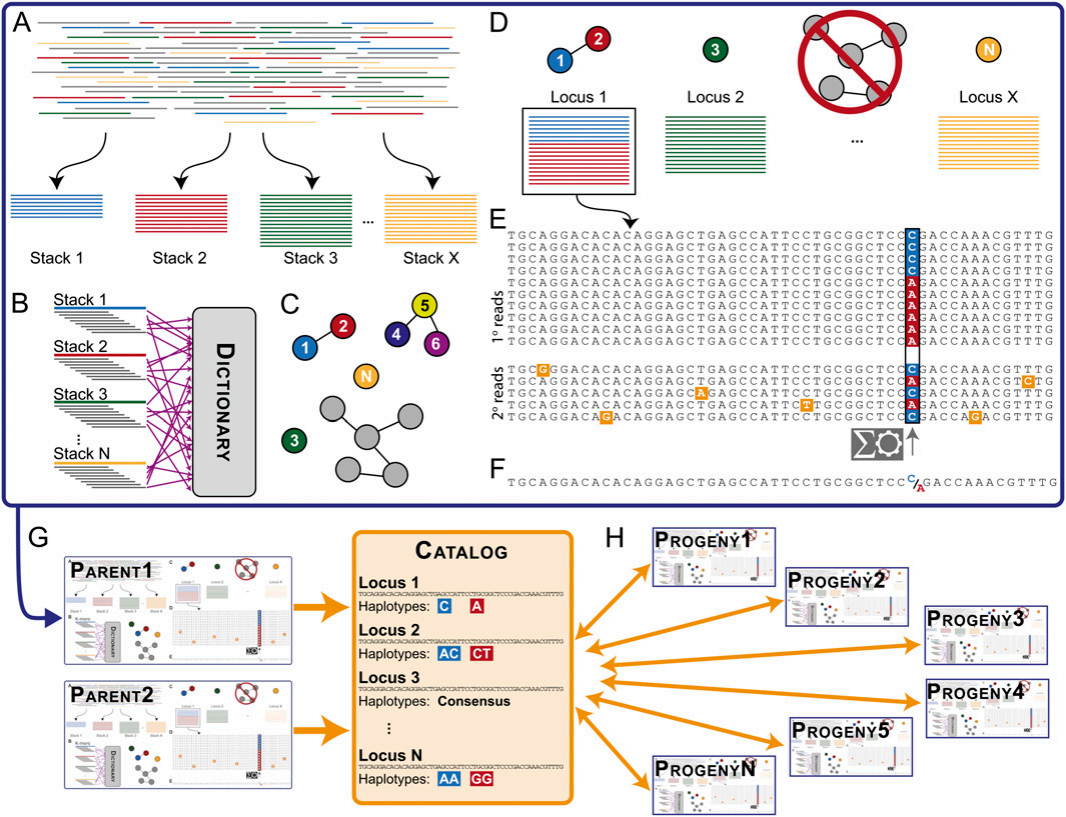
*Stacks* schematic. (A) The ustacks program forms stacks in an individual from short sequencing reads (cleaned by process_radtags.pl) that match exactly. (B) The ustacks program breaks down the sequence of each stack into k-mers and loads them into a dictionary. The ustacks program breaks down each stack again into k-mers and queries the k-mer Dictionary to create a list of potentially matching stacks, which can be visualized as nodes in a graph connected by the nucleotide distance between them. (C) ustacks merges matched stacks to form putative loci. (D) ustacks matches secondary reads that were not initially placed in a stack against putative loci to increase stack depth. An SNP model in ustacks checks each locus at each nucleotide position for polymorphisms. (E) ustacks calls a consensus sequence and records SNP and haplotype data. (F) The cstacks program loads stacks from the parents of a genetic cross into a Catalog to create a set of all possible loci in a mapping cross. (G) sstacks matches map cross progeny against the Catalog to determine the haplotypes at each locus in every individual in the cross.

The discussion here assumes that input to *Stacks* is composed of RAD-seq data (Figure 1A) from the parents and progeny of a genetic cross. *Stacks* builds map markers by identifying loci and their constituent alleles in each individual (Figure 1A–F) and by creating a Catalog of parental loci (Figure 1G). *Stacks* then matches progeny against the Catalog (Figure 1H), which defines alleles at each locus in each individual. At each stage, *Stacks* exports outputs into a MySQL database.

*Stacks* requires clean sequence data in FASTA or FASTQ output files (*e.g.*, Kelley *et al.* 2010) using the program process_radtags.pl. The process_radtags.pl program examines each read using a sliding window: if the average quality score within a window drops below 90% confidence [a Phred score of 10, Ewing and Green (1998)], *Stacks* discards the read. Thus, *Stacks* accepts reads with isolated errors but detects reads with prolonged drops in quality and discards them. Uncalled nucleotides, nonexistent barcodes, or deficient restriction enzyme cut sites can also cause *Stacks* to exclude reads. *Stacks* can correct isolated errors in the restriction cut site sequence or in the barcode if the barcode is two or more nucleotides distant in sequence space from other barcodes used in the same sequencing library.

#### Identifying stacks, inferring loci:

The ustacks (unique stacks) program reads cleaned sequences and distills data into unique, exactly matching stacks by loading reads into a hash table (Figure 1A). Unique stacks that contain fewer reads than a configurable threshold (the *stack-depth* parameter) are disassembled, and the reads are set aside because these stacks are indistinguishable from stacks generated with sequencing error. Reads in a stack are *primary reads*, and reads that are set aside are *secondary reads*. The ustacks program calculates the average depth of coverage, then identifies stacks that are two standard deviations above the mean and excludes them, along with all stacks that are one nucleotide apart from these extremely deep (*lumberjack*) stacks, which usually represent repetitive elements.

Polymorphic genetic loci produce stacks that differ in few nucleotides. A k-mer search algorithm defines loci based on a user-specified distance between stacks (the *within-individual distance* parameter). This configurable distance depends on the dataset’s genetic properties, such as polymorphism rate and read length, and usually allows just a few nucleotide differences. To implement this comparison, ustacks breaks the sequence of each stack into a set of overlapping fragments of equal length *k* (k-mers) (Edgar 2004; Vinga and Almeida 2003) (Figure 1B). The first k-mer spans nucleotides 1 to *k*, the second 2 to *k* + 1, the third 3 to *k* + 2, and so on. The ustacks program automatically maximizes k-mer length according to the allowed nucleotide difference (longer k-mer lengths produce less promiscuous k-mers that require fewer comparisons to other reads) and loads k-mers into the Dictionary (Figure 1B).

The ustacks program queries the k-mer Dictionary with each k-mer from each stack to identify other stacks with matching k-mers. For pairs of stacks with sufficient numbers of matching k-mers, ustacks aligns the pair, naively matching nucleotide by nucleotide to verify that each pair of stacks is within the allowable nucleotide distance, and if they are, it records a match.

The k-mer search algorithm transitively relates pairs of stacks. For example, if stacks 4 and 5 match and stacks 5 and 6 match with an allowable distance of one nucleotide, ustacks records two matching pairs (Figure 1C). Then ustacks merges all matching pairs, in this case merging 4, 5, and 6, even though 4 and 6 are two nucleotides apart. Merged stacks represent putative loci displayed as a graph with nodes representing unique stacks and edges weighted by the nucleotide distance between them (Figure 1C). In a full graph containing all stacks in the dataset, each putative locus represents a disconnected subgraph (Figure 1C).

In a diploid genetic cross, homozygous and heterozygous loci should contain one and two stacks, respectively. Allowing for some error, if more than three unique stacks have been merged, or if the coverage of the merged stack is more than two standard deviations above the mean coverage, ustacks shunts the stack to the deleveraging algorithm to determine which subset of these large stacks is most likely to represent a locus (see Appendix 1).

The process of merging stacks is iterative. With a user-specified distance of three nucleotides between stacks, ustacks first finds stacks that are a single nucleotide different and merges them, then continues at a distance of two, and finally at a distance of three. At the end of each round, ustacks excludes lumberjack stacks. Secondary reads (2° reads, Figure 1E) that were set aside earlier are now matched against putative loci using the k-mer search algorithm but with greater nucleotide distance (two nucleotides larger than the *within-individual distance* parameter by default). Secondary reads that do not have a best match to a unique defined locus are discarded. At the end of this stage, *Stacks* has constructed a set of putative loci from high-confidence unique stacks and has buttressed locus depth by adding secondary reads.

#### Inferring alleles and haplotypes:

The next step is to identify polymorphisms within loci. To detect polymorphisms and infer alleles (Figure 1E), ustacks examines each putative locus one nucleotide position at a time using a maximum likelihood framework (Hohenlohe *et al.* 2010) (see Appendix 1). Some loci have polymorphisms at more than one position, but rarely in a short-read locus would a recombination event occur between two polymorphisms; hence, the configuration of SNPs at a locus represents a haplotype.

SNPs and haplotypes are visualized as a two-dimensional matrix containing stacked sequencing reads (Figure 1E). *Stacks* identifies SNPs by examining the matrix column-wise and calls haplotypes by examining the matrix row-wise. Haplotypes that define alleles in each locus become genetic markers for subsequent analyses. Finally, *Stacks* determines a consensus sequence for each locus (Figure 1F).

#### Aggregating loci into a Catalog:

At this point, *Stacks* has constructed loci for one individual (the large top box, Figure 1A–F). After *Stacks* has accomplished this task for a number of individuals (*e.g.*, the two parents in a genetic cross), cstacks (Catalog stacks, Figure 1G) synthesizes a Catalog of loci that appear in members of the population.

The cstacks program reads the output from ustacks and merges loci into the Catalog. The first individual (say, the female parent of the cross) initializes the Catalog. Each additional individual is then merged into the Catalog in turn. Individual loci are matched to those already in the Catalog using the same k-mer search algorithm used by ustacks, except that each locus is represented in the k-mer dictionary by the set of k-mers resulting from each haplotype at that locus. When two loci match, cstacks merges their SNPs in the Catalog. If, however, those SNPs have conflicting alleles (for example, a fixed A in the Catalog and a segregating G/C in the locus that is being merged in), the merge fails and cstacks issues a warning. The cstacks program adjusts its haplotype calls based on the newly merged SNPs.

The *between-individual distance* parameter of cstacks allows for mismatches while merging loci into the Catalog. If each parent is fixed for a different allele at a particular locus, cstacks can detect the mismatch and properly merge the loci. This property is particularly useful when fixed differences occur, as in crosses between inbred populations or between divergent species.

#### Matching the population against the Catalog:

To identify which locus/haplotype combinations are present in each individual in the population, sstacks (search stacks) matches every individual in the cross, including the parents and the progeny, against the Catalog (Figure 1H). The sstacks program constructs a hash table from every haplotype in the Catalog, compares all haplotypes from an individual, and records matches. Loci that match more than one Catalog locus are excluded because their true matching locus in the Catalog is ambiguous; multiple loci, however, can still uniquely match the same Catalog tag (these could represent, for example, repetitive sequences in the progeny that are not in the parents); users can elect to exclude these in later analyses.

#### Calling mappable markers:

At this stage, *Stacks* has identified haplotypes segregating in each individual in the population. Next *Stacks* identifies informative markers. The markers.pl program identifies mappable markers in the parents by downloading Catalog matches from the MySQL database and tallying up all the matching parental haplotypes. The markers.pl program characterizes parental loci into 10 classes of mappable markers, including loci that are segregating in the family due to variation in a single parent (ab/–, two alleles, a and b, in one parent, and a missing restriction site in the second parent), loci homozygous within parents but heterozygous between parents (aa/bb), loci with two (ab/aa), three (ab/ac), or four (ab/cd) haplotypes, as well as other related types (Table 2).

**Table 2:** *Stacks* marker types

Marker type	Female	Male	Number of segregating alleles	Notes
ab/aa	Heterozygous	Homozygous	2	
aa/ab	Homozygous	Heterozygous	2	
ab/ab	Heterozygous	Heterozygous	2	
aa/bb	Homozygous	Homozygous	2	Detected by cstacks
ab/–	Heterozygous	Absent	2	Polymorphic RAD-site in male, restriction site mutated in female
–/ab	Absent	Heterozygous	2	Polymorphic RAD-site in female, restriction site mutated in male
ab/cc	Heterozygous	Homozygous	3	ab detected by ustacks, cc detected by cstacks
cc/ab	Homozygous	Heterozygous	3	ab detected by ustacks, cc detected by cstacks
ab/ac	Heterozygous	Heterozygous	3	
ab/cd	Heterozygous	Heterozygous	4	

*Stacks* has now processed enough data to genotype map cross progeny, but it first must build an index in the MySQL database to unify the outputs of the previous analyses. The index_radtags.pl program performs this task and provides results to the web interface. This business logic is implemented in the denovo_map.pl program, which executes ustacks, cstacks, sstacks, markers.pl, and index_radtags.pl, and then uploads data to the database.

At this point, *Stacks* calls genotypes from map cross progeny using genotypes.pl after specifying a particular map type (F1, F2, doubled haploid, or back cross) and an export type (JoinMap or R/qtl). The genotypes.pl program maps haplotypes in the progeny to the marker types detected in the parents. First, genotypes.pl downloads from the database the set of loci containing mappable markers recorded by markers.pl. It then maps haplotypes: if the first parent has haplotypes GA and AC, and the second parent has the GA haplotype, *Stacks* declares an ab/aa marker for this locus. The genotypes.pl program maps GA to a, and AC to b in the parents and checks progeny to see which haplotypes each contains, recording the genotypes (either ab or aa, in this case). Finally, genotypes.pl formats genotypes for use with the mapping program and outputs a properly formatted file. Users can specify the minimum number of matching progeny required for locus export.

#### Automated corrections:

Users can tell the genotypes.pl program to perform automated corrections for certain errors, including checking homozygous tags in the progeny to ensure that a SNP is not present. As described in Appendix 1, if the SNP model cannot identify a site as heterozygous or homozygous, the site is tentatively labeled a homozygote to facilitate matching to the Catalog in sstacks. If a second allele identified in the Catalog (*i.e.*, in the parents) is present in a progeny individual at a low frequency (less than 10% of reads in the stack), genotypes.pl corrects the genotype. Likewise, genotypes.pl removes a homozygous genotype call for a particular individual if the locus contains fewer than five reads supporting the genotype. Users can adjust these thresholds.

#### Iterative corrections:

The genotypes.pl program can optionally output a file formatted for loading into the database. The web interface allows users to manually correct genotypes. For example, a stack for Locus 1 in one of the progeny (Figure 1E) might have just one A allele but 19 C alleles. *Stacks* would call the genotype as homozygous C, not being able to distinguish the single A from a sequencing error. But if a homozygous C call results in a double cross-over involving this single locus, the genotype is more likely to be heterozygous C/A with the A allele undersequenced. Users can make this correction through the web interface, and the corrected genotype will be included on the next execution of genotypes.pl.

### Utilizing a reference genome

*Stacks* can identify loci not only *de novo* as described above but also using a reference genome. The two processes differ: instead of building stacks and loci from similar sequence reads, *Stacks* first aligns sequence reads to the reference genome using Bowtie (Langmead *et al.* 2009). And instead of invoking ustacks, we use pstacks (population stacks), which reads either Bowtie or SAM (Li *et al.* 2009) files and builds stacks based on alignment positions. SNP calling proceeds as before, and parameters exist for both cstacks and sstacks to build Catalog loci and to match against those loci, respectively, based on reference genome alignment positions instead of sequence distance. The business logic of this pipeline is embodied in the ref_map.pl program, which executes each stage and loads the resulting data into the database. Because pstacks and ustacks output the same file formats, the web interface displays them as in a genetic map.

### Generating paired-end mini-contigs and adding other sequence sets

Mini-contigs from Rad-seq paired-end reads can be assembled and added to *Stacks*, thereby providing several hundred additional genomic nucleotides downstream of each marker that increase hits to expressed sequence tags libraries and thus connect markers to protein coding genes in other organisms (Amores *et al.* 2011; Etter *et al.* 2011). The sort_read_pairs.pl program collates paired-end reads associated with each stack and outputs a FASTA file for each locus in the catalog. Users can execute a program such as Velvet (Zerbino and Birney 2008), which assembles reads in each FASTA file, to form contigs that can then be loaded into the *Stacks* MySQL database using the *Stacks* load_sequences.pl program.

The load_sequences.pl program assumes that the sequence definition line, which is preceded by a “greater than sign” (>) for each sequence in a FASTA file, is a Catalog locus ID, and will store that sequence in the MySQL database linked to the Catalog locus. Therefore, in addition to mini-contigs, if ESTs are available or were constructed *de novo* using RNA-seq (Mortazavi *et al.* 2008), they can also be loaded into the database after they are matched to catalog loci using a program such as Bowtie or BLAST (Altschul *et al.* 1997). Any sequence data loaded into the MySQL database can later be exported in association with their markers using export_catalog.pl.

In summary, the *Stacks* importing and exporting capabilities can associate *Stacks* markers with additional sequences, including mini-contigs and ESTs. These sequence sets can associate mappable loci in protein coding genes to orthologs in other species by BLAST searches, or to genomic contigs in an emerging reference genome.

### Web-based interface

**Figure 2:**
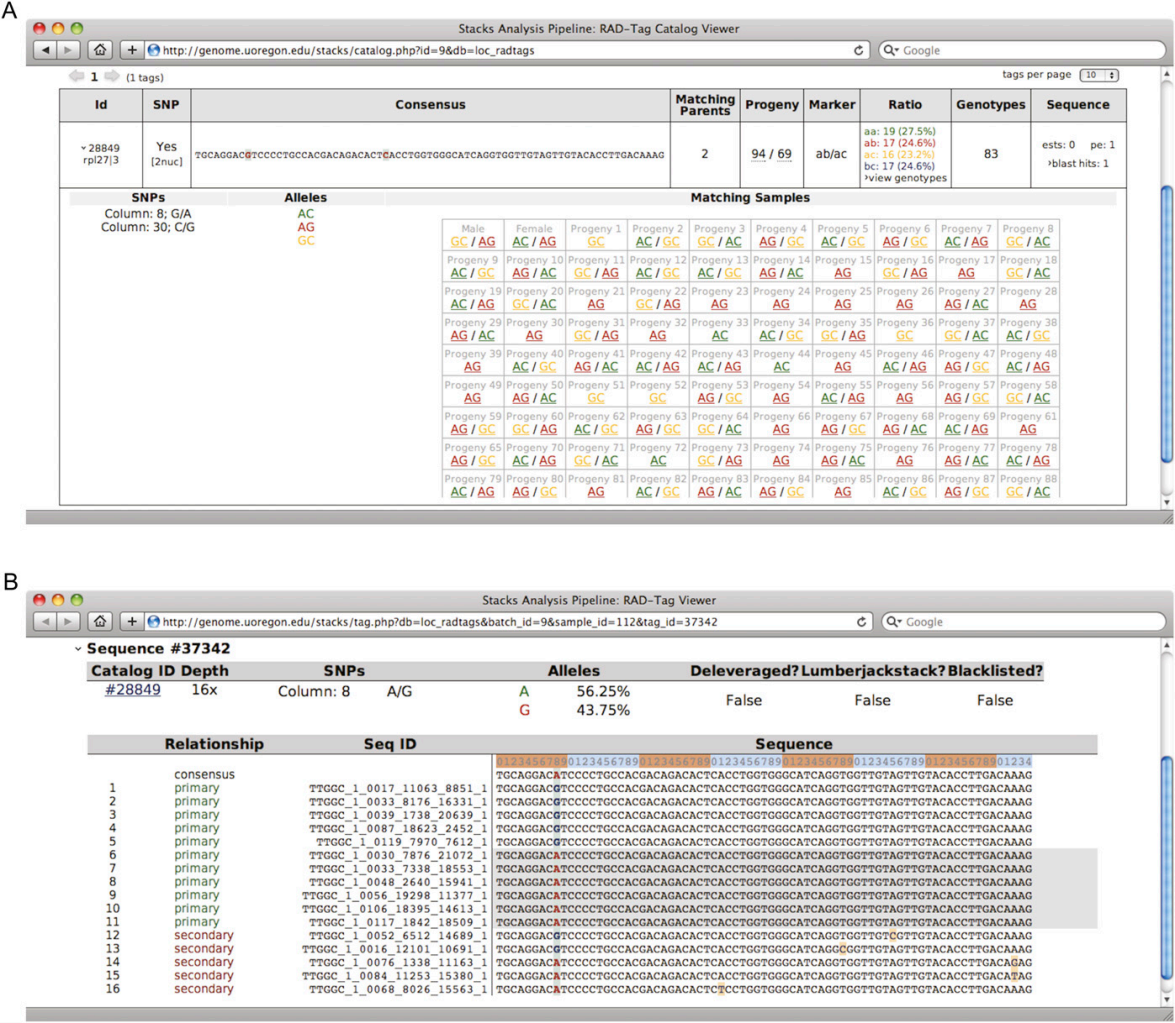
*Stacks* web interface. (A) The interface allows a researcher to view observed haplotypes at each locus in all individuals. (B) Researchers can click each haplotype to view the stack itself. The interface provides extensive filtering facilities as well as the ability to annotate and export results in a number of formats, including Excel, JoinMap, and R/qtl.

*Stacks* provides a web-based interface for viewing, annotating and correcting loci in a population (Figure 2). The web interface displays haplotypes present in every individual (Figure 2A) and clicking on a haplotype returns the appropriate stack (Figure 2B). The web interface, coupled with the MySQL database backend, provides extensive filtering capabilities, which facilitate the separation of useful data from background error, and it can export observed haplotypes as a Microsoft Excel document. This modular design allows *Stacks*, the database, and the web-based user interface to be located on the same or remote servers.

### Simulation results

**Figure 3:**
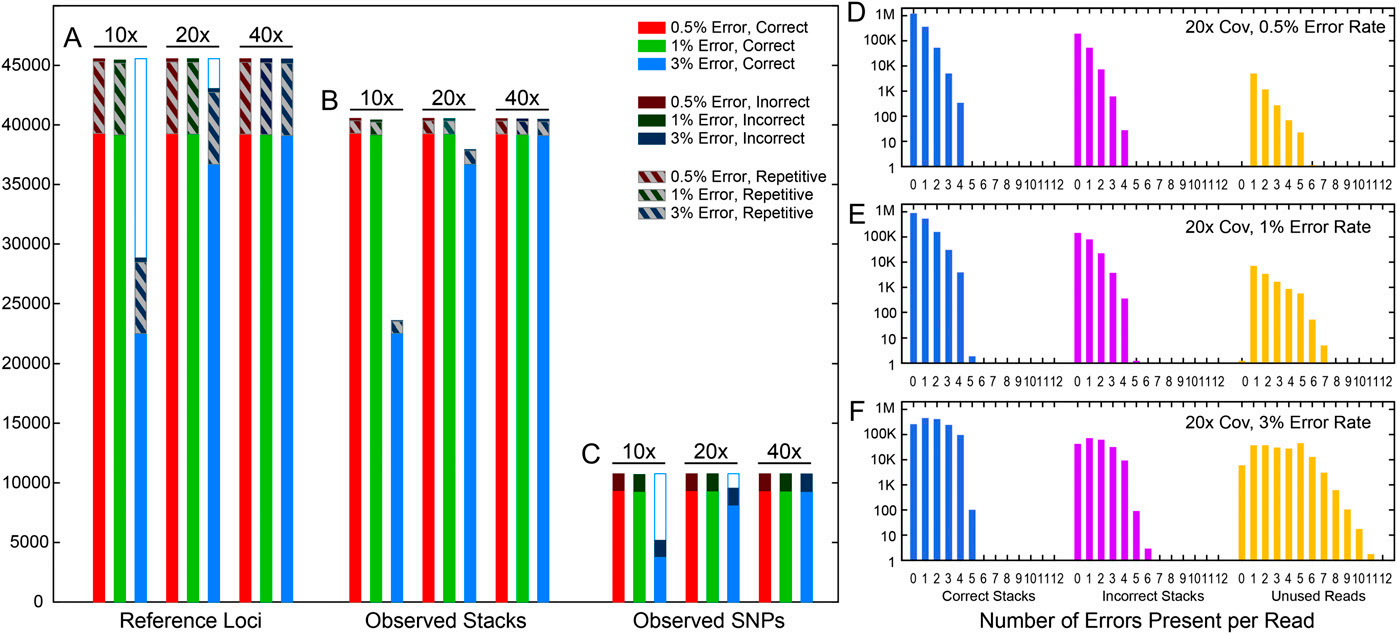
*Stacks* simulation results. The stickleback reference genome was digested *in silico* by *SbfI*, and 60 bp reads were made from each direction from the 22,774 cut sites at several different sequencing depths with several different error rates. The left panel shows the number of (A) loci, (B) stacks, and (C) SNPs observed in the *Stacks* output. Loci that *Stacks* assembled incorrectly are displayed in a dark color, whereas loci containing repetitive sequences are shown in a crosshatch pattern. A comparison of the number of loci present in the dataset (A) *vs.* the number of stacks reconstructed (B) showed that ustacks collapsed repetitive loci but correctly reconstructed nearly all other loci at low and moderate error rates or at high coverage. The right panel shows the number of reads with a certain number of sequencing errors that were incorporated into correct stacks, incorrect stacks, and unused reads for 20× coverage and error rates of (D) 0.5%, (E) 1%, and (F) 3%. As errors accumulated, *Stacks* excluded more reads, lowering the overall depth, whereas some reads accumulated enough errors to be incorporated into stacks that appeared to be correctly assembled but, in fact, joined stacks representing loci from which they did not originate (indicated by reads with more errors than allowed by the k-mer matching algorithm, four errors in the simulation).

To test the ability of ustacks to identify loci, we simulated the RAD-seq process from the well-assembled genome sequence of threespine stickleback. We generated data at a per-allele mean sequencing depth of 10×, 20×, and 40×, and we varied the sequencing error rate from 0.5 to 3%. In Figure 3, Reference Loci (Figure 3A) represents loci present in the stickleback reference genome (Ensembl version 59) after the RAD-seq simulation, whereas Observed Stacks (Figure 3B) represents data discovered by *Stacks*. Results showed that, at low and moderate error rates, ustacks correctly reconstructed nearly all (86%) known loci (Figure 3A, B). A comparison of Reference Loci and Observed Stacks, however (Figure 3A, B), shows that ustacks collapsed repetitive sequences. Apart from repetitive sequences, less than 1% of stacks assembled incorrectly. At the highest error rate (3%) and lowest coverage (10×), about 51% of the known loci disappeared from the results, but at 20× coverage, *Stacks* identified most loci (81% correctly assembled), and at 40× coverage, the error rate had little effect on the number of identified loci (86% correctly assembled, Figure 3A, B). Loci disappeared from the dataset likely due to low depth of coverage, which occurs by chance, as well as reads confounded by error.

The simulation further showed that ustacks robustly identified SNPs, except at a high error rate and low depth of coverage, or when confounded by repetitive sequences (Figure 3C). These data show that excess frugality or oversequencing are both wasteful. At the highest error rate and under the parameters of this simulation, moving from a per-allele depth of 10× to 20× gains 14,000 additional loci, whereas moving from 20× to 40×, which also doubles sequencing cost, nets only an additional 2400 loci.

To further study locus drop out and error rates, we examined the effect of error rate on the distribution of errors per read. Because our simulation allowed tracking the origin of each read, we could deduce that, at the lowest error rate, reads that ended up in either correct stacks or in incorrect stacks contained no more errors than are allowed by the k-mer matching algorithm (four errors, in the worst case) (Figure 3D). In contrast, reads that could not be assigned to a stack (unused reads) tended to have more errors even at the lowest error rate (Figure 3D). At higher error rates, the number of unused reads increased greatly, from approximately 6000 at 0.5% to about 200,000 at 3%, thus decreasing stack depth (Figure 3E, F). Reads with more errors than allowed by the matching algorithm (again, four errors) accumulated in all three categories of reads at a 3% error rate (Figure 3F). The accumulation of error-riddled reads in correctly assembled stacks indicates that some reads suffered enough error to make them more similar to a different locus than to their original, known locus. These results demonstrate that raw sequence quality has a strong effect on the ability of *Stacks* to successfully reconstruct loci.

**Figure 4:**
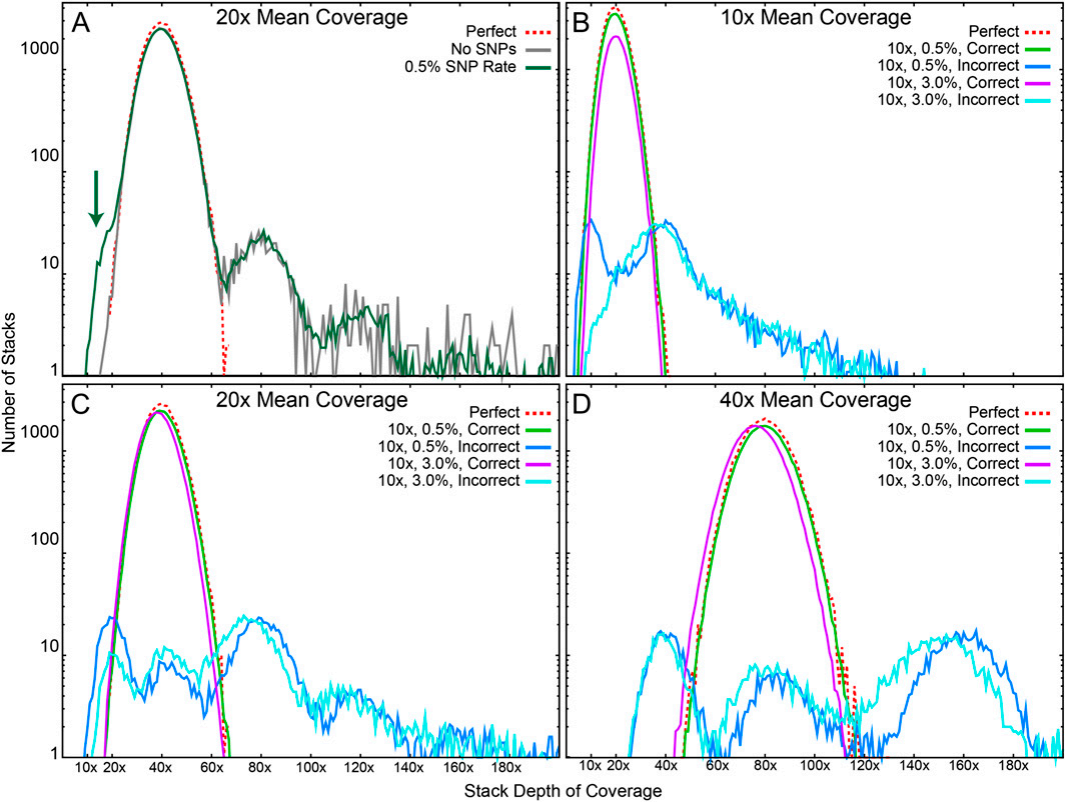
*Stacks* depth of coverage distribution. (A) Correctly reconstructed stacks have a depth of coverage equal to twice the mean sequencing coverage because the simulation assumes diploid individuals. With no polymorphism or error (gray line), the depth of coverage distribution nearly matched the known simulation distribution (dotted red line), with the exception of repetitive loci, which created the long tail of the distribution to the right, which was truncated at 200× but extends to 17,000×. After adding SNPs, ustacks failed to reconstruct a small number of loci (green arrow) as shown by the increase in stacks with a depth of coverage equal to the sequencing mean depth. (B–C) With the addition of sequencing error and increasing mean sequencing depth, most stacks were still properly reconstructed. Results showed a repeating pattern of improperly reconstructed stacks occurring at multiples of the mean sequencing depth corresponding to the number of loci improperly merged together. The increasing error rate caused a general loss of depth in the stacks (green *vs.* violet lines).

Simulation data revealed the interacting effects of SNPs, sequencing depth, and error rate on stack quality. First, consider the effect of introducing SNPs into simulated reads. The known distribution of stacks with a particular sequencing depth (Figure 4A, dotted red line) showed a peak at 40×, twice the average sequencing depth, because a diploid has two alleles at each locus. Without the introduction of SNPs or error, ustacks produced a rather erratic distribution of stack depth (Figure 4A, gray line) with peaks at 80× and 120× due to the erroneous collapsing of two or three loci known to be different because of their known origin in the stickleback genome. Furthermore, ustacks collapsed over 6000 repetitive *SbfI* RAD loci in the stickleback genome into a smaller number of loci with very high depths of coverage, as indicated by the long right tail of the distribution that stretches far beyond the truncated display in the figure. The introduction of SNPs into the simulated reads at a rate of 0.5% caused a shoulder to appear on the distribution at 20×, half the depth of the main peak (Figure 4A, green line). These erroneous stacks of approximately 20× depth appeared because ustacks failed to find and join the alternative alleles for these stacks.

To explore the effects of error rate on locus quality, we studied, at three levels of mean coverage, the effects of a typical low error rate of 0.5% and an unusually high error rate of 3.0% (Figure 4B, C). At 10× mean coverage and 0.5% error, the distribution of correctly formed loci matched closely that of the true distribution, differing only by having somewhat fewer loci (a 14% reduction) than actually exist (Figure 4B). This decrease came from two types of incorrectly joined stacks: some incorrect stacks occupied a peak at 10×, representing single stacks for which ustacks could not identify their true alternative alleles due to errors, and other incorrect stacks fell in a peak at 40×, representing cases in which ustacks inappropriately joined four stacks coming from two independent diploid loci. And still other stacks in the long tail represented the fusing of repetitive loci. An error rate six times higher (3%) reduced the number of correctly joined stacks to 49% of the true number and resulted in the loss of the peak at 10× found with the lower error rate. We conclude that high error rates cause inappropriate joining of stacks more frequently than incorrect failure to fuse stacks. With the introduction of errors at sequencing depths of 20× and higher, the distribution of correctly joined stacks shifted slightly to the left due to the accumulation of unused reads (Figure 4C, D, green *vs.* purple lines). In sum, these simulations demonstrate remarkable fidelity of locus identification, even in the face of mounting errors, when the sequencing depth is between 20× and 40×.

### A zebrafish genetic map

**Figure 5:**
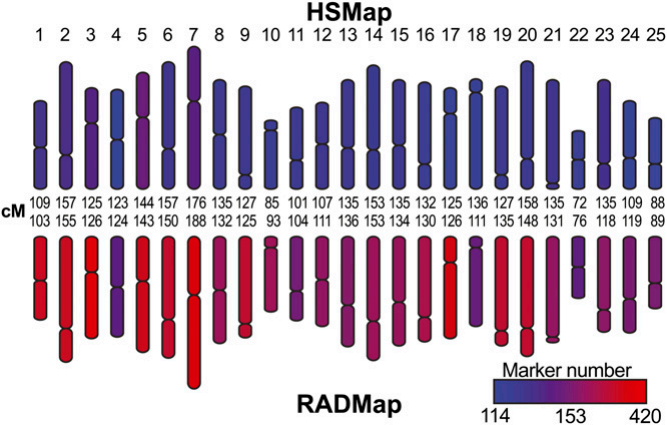
*Danio rerio* RAD-tag map compared to the doubled haploid map. We constructed a RAD-seq genetic map of zebrafish (RADmap) using DNA from 42 individuals of the doubled haploid mapping panel (HSmap) that had been previously genotyped by microsatellites or single strand conformation polymorphism (Kelly *et al.* 2000; Woods *et al.* 2000; Woods *et al.* 2005). *Stacks* recovered the 25 zebrafish linkage groups (Figure S2) with lengths nearly identical to published values (3186 cM in the HSmap *vs.* 3160 cM in the RADmap). With 7861 markers, our RADmap had nearly twice as many markers as appeared in the HSmap (4073 markers). The insert shows the scale for marker density.

If *Stacks* works well, it should reconstruct a known genome map. To test this prediction, we constructed for *Danio rerio* a genetic map (RADmap) by using RAD-seq and *Stacks* to re-genotype a previously published doubled haploid mapping panel (HSmap, http://zfin.org/cgi-bin/webdriver?MIval=aa-crossview.apg&OID=ZDB-REFCROSS-000320-1) that consists of 42 progeny (Kelly *et al.* 2000; Woods *et al.* 2005). *Stacks* reconstructed the 25 zebrafish linkage groups (Figure S2), each with a length nearly identical to the original (Figure 5, 3186 cM in the HSmap *vs.* 3160 cM in the RADmap). With 7861 markers, our RADmap has nearly twice as many markers as the original HSmap (4073 markers), but it required less than 1% of the cost and took less than 1% of the time to genotype and construct. The RADmap and HSmap had nearly identical marker order (Figure S3); differences could represent errors in either map.

**Figure 6:**
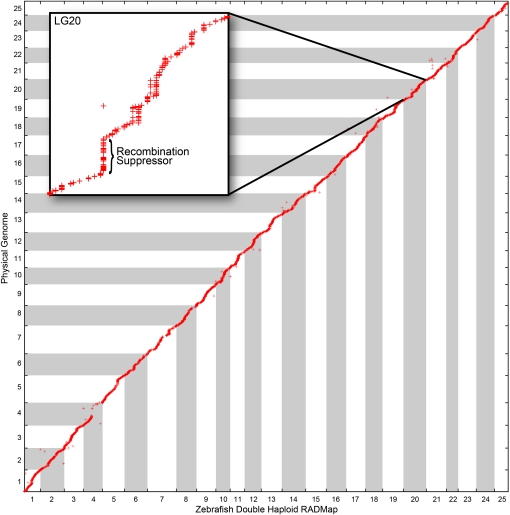
RADmap marker order is consistent with the sequenced zebrafish genome. A specific region on LG20 with no recombination in the RADmap spanned almost 10 Mb in the physical genome (inset). This recombination suppression could be due to a heterozygous inversion present in the genome of the mother of the gynogenetic HS mapping panel.

A comparison of the zebrafish RADmap to the sequenced reference genome showed alignment of 5787 RADmap markers and revealed that marker order for the RADmap and the physical assembly generally agreed (Figure 6). An additional 157 mapped RADmap markers aligned to genomic scaffolds that are currently unordered in the Zv9 reference genome, thus positioning these errant contigs into the reference genome.

A plot of the RADmap *vs.* the reference genome identified several regions of low recombination rate per physical distance. One region in LG20 showed recombination suppression in the RADmap over a region of about 10 Mb (Figure 6, inset) that could be due to a heterozygous inversion in the mother of the HS mapping panel, who was a heterozygote of the clonal C32 line and the highly inbred SJD strains (Nechiporuk *et al.* 1999; Streisinger *et al.* 1986). This hypothesis, generated by the extraordinarily high density of RAD markers, warrants further investigation. LG4, which is chromosome 3 in the physical genome (Phillips *et al.* 2006), has a mostly heterochromatic long arm, whose repetitive elements would produce lumberjack stacks that would be excluded from analysis. Markers off the diagonal of Figure 5 could be due to errors either in the RADmap, in the BLAST assignment of RADmap markers to the physical genome, or in the physical assembly. These results show that RAD-tag markers can recapitulate a known genetic map at greater density and with less time and expense than methodologies currently in use.

## Discussion

Analyzing RAD-seq data with *Stacks* can recover hundreds to tens of thousands of informative markers that describe the genetics of a population. *Stacks* has been used to generate an ultradense genetic map using the F1 offspring of wild-caught spotted gar (Amores *et al.* 2011), to examine the phylogeographic distribution of the mosquito, *Wyeomyia smithii* (Emerson *et al.* 2010), and to generate informative SNPs in trout populations (Hohenlohe *et al.* 2011). The zebrafish map constructed *de novo* here and compared with a well-assembled sequenced genome demonstrates the rapid nature of this approach that took a few weeks of part-time effort, whereas a previous map using the same DNAs required several years to construct, cost 100 times as much, and had half the number of markers.

Having shown the biological precision of *Stacks*, we now discuss how to increase its informative value by iteratively improving data and associating loci to additional sequence data. Appendix 2 presents methods to optimize *Stacks*, including alternative strategies to build the Catalog and to adjust important *Stacks* parameters.

### *Stacks* reveals loci *en mass*

Not all RAD-seq loci appear in all individuals due to polymorphisms in restriction enzyme cut sites, stochastic events related to sequencing (as our simulations showed), PCR errors, or sequencing errors. Loci that appear in a large number of individuals in a population or in a large number of map cross progeny are the most reliable. Once *Stacks* has generated a set of markers, it is most effective to select markers supported in as many progeny as possible by using the set of filters provided in the web interface (Figure 2) or by specifying a minimum number of progeny when exporting genotypes.

### Iterative corrections

One of the key attributes of *Stacks* is its convenient web interface, which supports manual corrections. Iterative corrections can make significant improvements in a genetic map based on the principle that double recombinants in a short genetic distance are unlikely events. Manual examination of markers that expand the map can identify, correct, or remove troublesome genotypes, followed by re-exporting data and reconstructing the map. Reiteration can provide a genetic map with strong statistical support on all linkage groups.

### *Stacks* and genome duplication

Genome duplication events, like those that occurred in the stems of vertebrate, teleost, salmonid, and flowering plant lineages (Allendorf and Danzmann 1997; Amores *et al.* 1998; Dehal and Boore 2005; Koop *et al.* 2008; Jiao *et al.* 2011), result in paralogs that are initially identical but diverge over time. In some cases, *Stacks* might erroneously confuse paralogs that have nearly identical sequences with alleles of the same locus. Fortunately, *Stacks* can detect “overmerging” of paralogous stacks because all individuals homozygous for a specific sequence at one paralog and homozygous for a slightly different sequence in the other paralog would appear to be heterozygotes for the relevant SNP. In contrast, a meiotic mapping population that is segregating a SNP at one locus or a population in Hardy-Weinberg equilibrium would have, on average, only about half of the individuals being heterozygotes. In addition, a diploid individual will never have more than two alleles of a single locus, so if individuals are discovered with three or more alleles, paralogs are likely to blame. *Stacks* can detect markers in which observed heterozygosity is significantly different than expected and flag them. The problem of confusing paralogs with allelic variants is evolutionarily transitory. Identical stacks (as might be found for paralogs in recent tetraploids) are uninformative and don’t cause a problem; furthermore, a few neutral mutations are sufficient for *Stacks* to identify paralogous loci, particularly if the user sets the *within-distance* parameter to a small value. In a recent study of trout populations, *Stacks* flagged loci that differed from Hardy-Weinberg expectations, thereby successfully removing the effects of the recent (25–100 million years ago) salmonid genome duplication (Hohenlohe *et al.* 2011).

The stringency of applied filters should depend on a number of factors that reflect both the biology of the species (*e.g.*, time since duplication) and the experimental goals (*e.g.*, trade-off between marker number and marker reliability). In some cases, however, the indiscriminate filtering of loci that do not appear to meet Hardy-Weinberg expectations can lead to erroneous conclusions. For example, in a recent moss linkage map, 45% of the loci exhibited segregation distortion, likely due to lethal interactions between distant loci (Mcdaniel *et al.* 2007). Thus, while *Stacks* can flag markers that do not fit expectations, careful interpretation is required to understand the biology of the species.

### Increasing the informative value of *Stacks*

Given the high marker density of a RAD-seq genetic map and the fact that those markers consist of genomic sequence, BLAST searches can associate markers or mini-contigs to ESTs, such as those generated by RNA-seq, or to orthologous genes in other species (Amores *et al.* 2011). These features make comparative genomics a natural extension of a *Stacks* analysis. The *Stacks* database contains several tables supporting the importation of paired-end mini-contigs or RNA-seq-assembled ESTs. A table also exists to store BLAST hits from markers, mini-contigs, or ESTs, and the web interface displays these data. Combined with programs in *Stacks* that import and export these sequences from the database, it becomes straightforward to perform conserved synteny analyses on genetic maps (see Amores *et al.* 2011). Mini-contigs can be exported to help design PCR primers for marker-assisted selection or to isolate genomic clones for specific markers in the genetic map. In addition, *Stacks* facilitates the alignment of genomic contigs from an emerging, often highly fragmented, reference genome assembly to the genetic map, thereby creating linkage group–based scaffolds from the physical contigs.

Nearly a century after the first genetic maps (Sturtevant 1913), *Stacks*, coupled with massively parallel DNA sequencing, makes the genetic map relevant again. Because *Stacks* and RAD-seq rapidly and inexpensively provide unprecedented numbers of genetic markers, fragmented genome assemblies can be ordered, and variation existing in single individuals taken directly from the wild can provide genetic maps with genome-wide comparative information. In addition, *Stacks* makes genome-wide association studies (GWAS) more tractable in nonmodel species because the enormous linkage map provides a framework for the analysis of population genomic data. *Stacks* is available for download, along with a set of example data, tutorials, and other documentation at http://creskolab.uoregon.edu/stacks/.

## Supplementary Material

Supporting Information

## Appendix 1

### ALGORITHMIC DETAILS

#### The deleveraging algorithm

Given a connected graph of unique stacks, the deleveraging algorithm contained in ustacks determines which subset of stacks is most likely to represent a locus. This heuristic program assumes that stacks originating from the same locus have approximately the same depth of coverage; for example, a true stack should have greater depth of coverage than stacks resulting from sequencing errors but less depth than stacks derived from repetitive elements. To achieve the goal of identifying the true components of the locus, the deleveraging algorithm first scales edges in the graph (which reflect nucleotide distance) by the log of the difference in depth of coverage between nodes. Nodes with a small nucleotide distance and relatively equal depths of coverage will be connected by an edge weighted with a very small distance in the graph, whereas nodes separated by a large nucleotide distance and/or a large difference in depth of coverage will be connected by an edge weighted by a large distance. *Stacks* feeds these scaled distances into a hierarchical clustering algorithm (de Hoon 2010) that arranges stacks in a tree according to distances that separate stacks from one another. This resulting tree is split into two groups, one representing the most closely related nodes in the tree, and the other containing the rest. For example, given a set of five stacks that have been grouped into a putative locus, where two stacks have coverage depth similar to that of the mean and where the other three stacks are shallow due to sequencing errors, the deleveraging algorithm is successful if it can separate the two real stacks from the stacks with sequencing errors. After the deleveraging algorithm executes, *Stacks* checks the maximum distance between the nodes in both clusters, and if this distance is greater than the user-specified nucleotide distance (the *within-individual distance* parameter), the lumberjack stack is blacklisted and excluded from use in the remainder of the pipeline; otherwise, the grouped loci persist in the dataset.

#### Maximum likelihood SNP model

At each nucleotide position in a locus, *Stacks* adapts the single-nucleotide, diploid genotyping method described by (Hohenlohe *et al.* 2010). This approach considers the counts of each of the four possible nucleotides in a multinomial sampling and uses a likelihood ratio test to assess the significance of the most likely genotype. The sequencing error rate is estimated implicitly by maximum likelihood at each position, and a significance level of α = 0.05 is used (without correction for multiple testing) to assign a diploid genotype (homozygote or heterozygote) at each position for each individual (Hohenlohe *et al.* 2010). If this likelihood ratio test is not significant, due either to low coverage or to read counts that lie between expectations for a heterozygote and a homozygote with error, then the model considers the location to be a homozygote for the most commonly observed nucleotide. This procedure avoids uncalled bases in the subsequent merging of stacks. The genotypes.pl program later corrects these genotypes by using information across individuals in the dataset. Future versions of *Stacks* will allow prior distributions to be placed on the sequencing error parameter and the significance level α to accommodate known attributes of the dataset, and it will dynamically modify thresholds that determine when a particular genotype call is significant.

## Appendix 2

### IMPORTANT PARAMETERS; USAGE STRATEGIES

#### Catalog construction

The cstacks program, which builds the Catalog, can accept data from an arbitrary number of individuals. The Catalog was designed to contain all possible loci that might appear in an analysis. The merging of each additional individual into the Catalog, however, brings with it a small number of erroneous stacks. In the case of a genetic map, the choice of what to load into the Catalog is simple: the parents of the cross contain all possible loci present in the progeny and thus together act as a natural limit to the number of loci that will be in their progeny; hence, the parents should appear in the Catalog. In a population genomic investigation, however, loading individuals into the Catalog is likely to increase the number of erroneous loci in the Catalog as a function of the number of individuals loaded. One strategy around this problem is to create a “superparent,” a virtual individual created by combining reads from many individuals. The ustacks program builds stacks from the superparent normally, and cstacks loads them into the Catalog. If the investigated individuals are members of different subpopulations (say, lake *vs.* marine fish or different plant ecotypes), then a superparent could be built from each subpopulation with several superparents loaded into the Catalog. This approach works well for correctly identifying loci with a moderate to high frequency of minor alleles in the population sample, which is likely to be a primary application of *Stacks*. Note, however, that the multinomial sampling model in the genotyping algorithm applies strictly to single diploid individuals, where alternative alleles in a single heterozygous individual are expected to appear in a stack in roughly equal frequencies. Rare alleles in a population might be erroneously excluded by this approach because the program treats their SNPs as sequencing errors in the superparent. In the future, we plan to eliminate stacks representing errors by comparing loci across a population and eliminating very low frequency haplotypes at a particular locus, which may obviate the need to construct superparents.

#### Important parameter values

Users can specify three major parameters when running the *Stacks* pipeline *de novo*.

The *stack-depth* parameter controls the minimum number of identical reads required to form a stack. In our empirical work, we have had success using a minimum depth of three, although this number should scale along with the number of raw reads available.

The second parameter, the *within-individual distance* parameter, is the maximum number of nucleotide mismatches allowed between stacks before fusing two or more stacks into a locus. If this parameter is set too low, loci containing multiple SNPs per haplotype will not be recovered. If it is set too high, *Stacks* will incorrectly combine distinct genetic loci that happen to be near each other in sequence space. The default value is two nucleotides, but we have had success using a value of up to four. This parameter must be sensitive to the biology (for example, duplication history) of the species.

The third parameter, called the *between-individual distance* parameter, is the number of mismatches allowed between loci in the Catalog. The *between-individual distance* parameter allows *Stacks* to detect loci that are homozygous in individuals but polymorphic between individuals. By default, *Stacks* sets catalog-mismatch to zero; increasing the catalog-mismatch limit can have the same potentially negative effects as increasing the *within-individual distance* parameter. The most appropriate value for the *between-individual distance* parameter varies with the evolutionary distance of the parents of the cross or of the members of the population being examined. In a standard F2 mapping cross, an F1 pseudo-testcross [as in the gar map (Amores *et al.* 2011)], or the zebrafish doubled haploid cross, zero is appropriate for the *between-individual distance* parameter. In contrast, with highly divergent populations, a higher value might be more appropriate because different populations might be fixed for different alleles of the same locus. A reasonable approach is to set the *between-individual distance* parameter to the same value as the *within-individual distance* parameter if one expects fixed alleles in the parents of a cross or in members of a population.

## References

[bib1] Allendorf F. W. Danzmann R. G., 1997 Secondary tetrasomic segregation of MDH-B and preferential pairing of homeologues in rainbow trout. Genetics 145: 1083–1092 9093860 10.1093/genetics/145.4.1083 PMC1207878

[bib2] Altschul S. F. Madden T. L. Schaffer A. A. Zhang J. Zhang Z., 1997 Gapped BLAST and PSI-BLAST: a new generation of protein database search programs. Nucleic Acids Res. 25: 3389–3402 9254694 10.1093/nar/25.17.3389 PMC146917

[bib3] Amores A. Force A. Yan Y. L. Joly L. Amemiya C., 1998 Zebrafish hox clusters and vertebrate genome evolution. Science 282: 1711–1714 9831563 10.1126/science.282.5394.1711

[bib4] Amores A. Catchen J. M. Ferrara A. Fontenot Q. Postlethwait J. H., 2011 Genome evolution and meiotic maps by massively parallel DNA sequencing: spotted gar, an outgroup for the teleost genome duplication. Genetics 188: 799–808 21828280 10.1534/genetics.111.127324 PMC3176089

[bib5] Arias J. Keehan M. Fisher P. Coppieters W. Spelman R., 2009 A high density linkage map of the bovine genome. BMC Genet. 10(1): 18 19393043 10.1186/1471-2156-10-18 PMC2680908

[bib6] Baird N. A. Etter P. D. Atwood T. S. Currey M. C. Shiver A. L., 2008 Rapid SNP discovery and genetic mapping using sequenced RAD markers. PLoS ONE 3(10): e3376 18852878 10.1371/journal.pone.0003376 PMC2557064

[bib7] Broman K. W. Wu H. Sen Ś. Churchill G. A., 2003 R/qtl: QTL mapping in experimental crosses. Bioinformatics 19(7): 889–890 12724300 10.1093/bioinformatics/btg112

[bib8] de Hoon M. J. L., 2010 The C Clustering Library for cDNA microarray data. Available at: http://bonsai.hgc.jp/∼mdehoon/software/cluster/software.htm#source

[bib9] Dehal P. Boore J. L., 2005 Two rounds of whole genome duplication in the ancestral vertebrate. PLoS Biol. 3(10): e314 16128622 10.1371/journal.pbio.0030314 PMC1197285

[bib10] Edgar R., 2004 Local homology recognition and distance measures in linear time using compressed amino acid alphabets. Nucleic Acids Res. 32(1): 380–385 14729922 10.1093/nar/gkh180 PMC373290

[bib11] Emerson K. J. Merz C. R. Catchen J. M. Hohenlohe P. A. Cresko W. A., 2010 Resolving postglacial phylogeography using high-throughput sequencing. Proc. Natl. Acad. Sci. U S A 107(37): 16196–16200 20798348 10.1073/pnas.1006538107 PMC2941283

[bib12] Etter P. D. Preston J. L. Bassham S. Cresko W. A. Johnson E. A., 2011 Local *de novo* assembly of RAD paired-end contigs using short sequencing reads. PLoS ONE 6(4): e18561 21541009 10.1371/journal.pone.0018561 PMC3076424

[bib13] Ewing B. Green P., 1998 Base-calling of automated sequencer traces using Phred. II. Error probabilities. Genome Res. 8(3): 186–194 9521922

[bib14] Hohenlohe P. A. Amish S. J. Catchen J. M. Allendorf F. W. Luikart G., 2011 Next-generation RAD sequencing identifies thousands of SNPs for assessing hybridization between rainbow and westslope cutthroat trout. Molecular Ecology Resources 11: 117–122 21429168 10.1111/j.1755-0998.2010.02967.x

[bib15] Hohenlohe P. A. Bassham S. Etter P. D. Stiffler N. Johnson E. A., 2010 Population genomics of parallel adaptation in threespine stickleback using sequenced RAD tags. PLoS Genet. 6(2): e1000862 20195501 10.1371/journal.pgen.1000862 PMC2829049

[bib16] Jiao Y. Wickett N. J. Ayyampalayam S. Chanderbali A. S. Landherr L., 2011 Ancestral polyploidy in seed plants and angiosperms. Nature 473: 97–100 21478875 10.1038/nature09916

[bib17] Kelley D. Schatz M. Salzberg S., 2010 Quake: quality-aware detection and correction of sequencing errors. Genome Biol. 11(11): R116 21114842 10.1186/gb-2010-11-11-r116 PMC3156955

[bib18] Kelly P. D. Chu F. Woods I. G. Ngo-Hazelett P. Cardozo T., 2000 Genetic linkage mapping of zebrafish genes and ESTs. Genome Res. 10(4): 558–567 10779498 10.1101/gr.10.4.558 PMC310859

[bib19] Koop B. F. von Schalburg K. R. Leong J. Walker N. Lieph R., 2008 A salmonid EST genomic study: genes, duplications, phylogeny and microarrays. BMC Genomics 9: 545 19014685 10.1186/1471-2164-9-545 PMC2628678

[bib20] Langmead B. Trapnell C. Pop M. Salzberg S., 2009 Ultrafast and memory-efficient alignment of short DNA sequences to the human genome. Genome Biol. 10(3): R25 19261174 10.1186/gb-2009-10-3-r25 PMC2690996

[bib21] Li H. Handsaker B. Wysoker A. Fennell T. Ruan J., 2009 The Sequence Alignment/Map format and SAMtools. Bioinformatics 25(16): 2078–2079 19505943 10.1093/bioinformatics/btp352 PMC2723002

[bib22] McDaniel S. F. Willis J. H. Shaw A. J., 2007 A linkage map reveals a complex basis for segregation distortion in an interpopulation cross in the moss Ceratodon purpureus. Genetics 176: 2489–2500 17603096 10.1534/genetics.107.075424 PMC1950648

[bib23] Miller M. R. Dunham J. P. Amores A. Cresko W. A. Johnson E. A., 2007 Rapid and cost-effective polymorphism identification and genotyping using restriction site associated DNA (RAD) markers. Genome Res. 17(2): 240–248 17189378 10.1101/gr.5681207 PMC1781356

[bib24] Mortazavi A. Williams B. A. McCue K. Schaeffer L. Wold B., 2008 Mapping and quantifying mammalian transcriptomes by RNA-Seq. Nat. Methods 5(7): 621–628 18516045 10.1038/nmeth.1226 PMC13303166

[bib25] Nechiporuk A. Finney J. E. Keating M. T. Johnson S. L., 1999 Assessment of polymorphism in zebrafish mapping strains. Genome Res. 9(12): 1231–1238 10613846 10.1101/gr.9.12.1231 PMC311009

[bib26] Phillips R. B. Amores A. Morascha M. R. Wilson C. Postlethwait J. H., 2006 Assignment of zebrafish genetic linkage groups to chromosomes. Cytogenet. Genome Res. 114(2): 155–162 16825768 10.1159/000093332

[bib27] Postlethwait J. Johnson S. Midson C. Talbot W. Gates M., 1994 A genetic linkage map for the zebrafish. Science 264(5159): 699–703 8171321 10.1126/science.8171321

[bib28] Shimoda N. Knapik E. W. Ziniti J. Sim C. Yamada E., 1999 Zebrafish genetic map with 2000 microsatellite markers. Genomics 58(3): 219–232 10373319 10.1006/geno.1999.5824

[bib29] Snyder M. Du J. Gerstein M., 2010 Personal genome sequencing: current approaches and challenges. Genes Dev. 24(5): 423–431 20194435 10.1101/gad.1864110 PMC2827837

[bib30] Streisinger G. Singer F. Walker C. Knauber D. Dower N., 1986 Segregation analyses and gene-centromere distances in zebrafish. Genetics 112(2): 311–319 3455686 10.1093/genetics/112.2.311 PMC1202703

[bib31] Sturtevant A. H., 1913 The linear arrangement of six sex-linked factors in Drosophila, as shown by their mode of association. J. Exp. Zool. 14: 43–59

[bib32] Sun Z. Wang Z. Tu J. Zhang J. Yu F., 2007 An ultradense genetic recombination map for Brassica napus, consisting of 13551 SRAP markers. TAG Theoretical and Applied Genetics 114: 1305–1317 10.1007/s00122-006-0483-z 17426959

[bib33] Van Ooijen J. W., 2006 JoinMap 4.0: Software for the Calculation of Genetic Linkage Maps in Experimental Populations. Kyazma B.V., Wageningen, Netherlands

[bib34] van Os H. Andrzejewski S. Bakker E. Barrena I. Bryan G. J., 2006 Construction of a 10,000-marker ultradense genetic recombination map of potato: providing a framework for accelerated gene isolation and a genomewide physical map. Genetics 173(2): 1075–1087 16582432 10.1534/genetics.106.055871 PMC1526527

[bib35] Vinga S. Almeida J., 2003 Alignment-free sequence comparison - a review. Bioinformatics 19(4): 513–523 12611807 10.1093/bioinformatics/btg005

[bib36] Woods I. G. Kelly P. D. Chu F. Ngo-Hazelett P. Yan Y.-L., 2000 A comparative map of the zebrafish genome. Genome Res. 10(12): 1903–1914 11116086 10.1101/gr.10.12.1903 PMC313070

[bib37] Woods I. G. Wilson C. Friedlander B. Chang P. Reyes D. K., 2005 The zebrafish gene map defines ancestral vertebrate chromosomes. Genome Res. 15(9): 1307–1314 16109975 10.1101/gr.4134305 PMC1199546

[bib38] Zerbino D. R. Birney E., 2008 Velvet: algorithms for de novo short read assembly using de Bruijn graphs. Genome Res. 18(5): 821–829 18349386 10.1101/gr.074492.107 PMC2336801

