# Supplementary material for: Stacks: Building and Genotyping Loci De Novo From Short-Read Sequences
Source: G3 (Bethesda). 2011 Aug 1;1(3):171–82. doi: 10.1534/g3.111.000240 (PMC3276136; doi:10.1534/g3.111.000240)
Supplement: Supporting Information [file supp_1_3_171__index.html]

Supporting Information 

# *Stacks*: Building and Genotyping Loci *De Novo* From Short-Read Sequences

## Supporting Information for Catchen *et al.*, 2011

**Files in this Data Supplement:**

- Supporting Information - Figures S1-S3 (PDF, 316 KB)
- Figure S1 - RAD-seq Simulation (PDF, 316 KB)
- Figure S2 - *Danio rerio* RADmap (PDF, 316 KB)
- Figure S3 - HSmap versus RADmap (PDF, 316 KB)
