# Supplementary material for: Stacks: Building and Genotyping Loci De Novo From Short-Read Sequences
Source: G3 (Bethesda). 2011 Aug 1;1(3):171–82. doi: 10.1534/g3.111.000240 (PMC3276136; doi:10.1534/g3.111.000240)
Supplement: Supporting Information [file supp_1.3.171_000240SI.pdf]

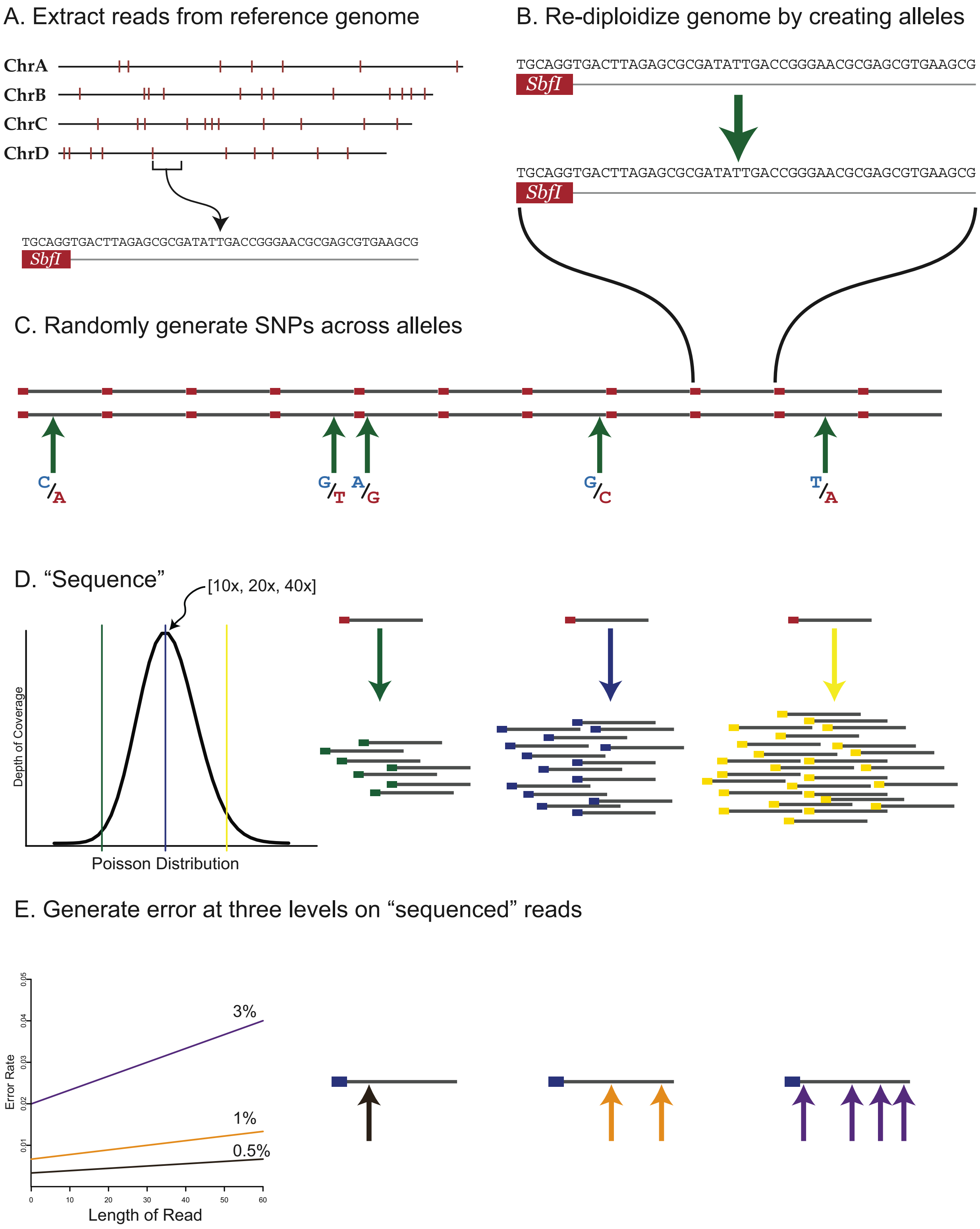

Figure S1 RAD-seq Simulation. (A) 60bp reads were extracted *in silico* from the stickleback reference genome at each occurrence of an *SbfI* restriction enzyme cut site. (B) Extracted reads were re-diplodized and (C) SNPs were added to the reads at a uniform rate of 0.5%. (D) The reads were "sequenced" at per-allele mean depths of 10x, 20x, and 40x by drawing numbers from a Poisson distribution. (E) Errors were added to the "sequenced" reads at three rates, 0.5%, 1%, and 3%.

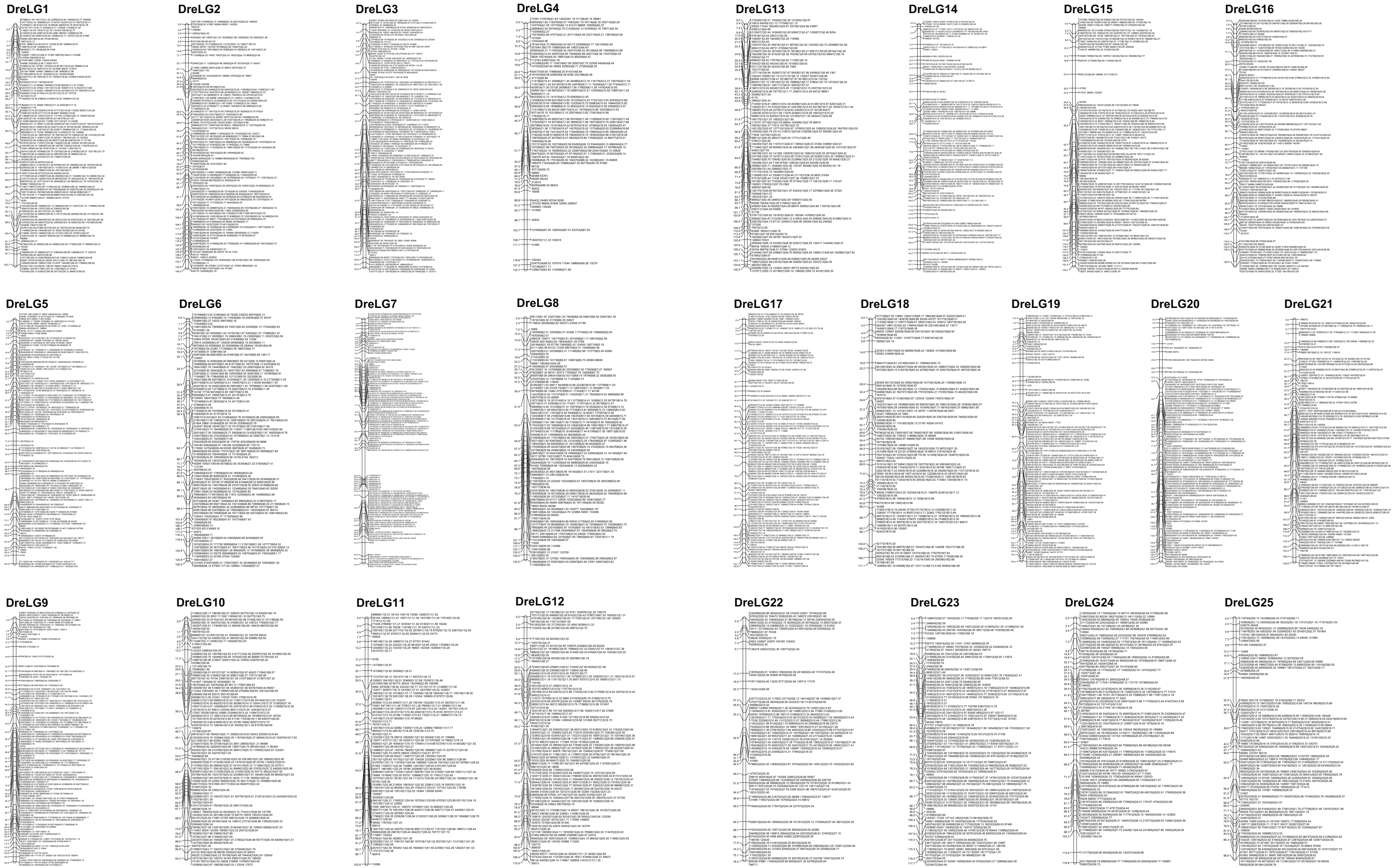

Figure S2 *Danio rerio* RADmap. We mapped 7,861 markers generated from a doubled haploid mapping panel; analysis by *Stacks* recapitulated the 25 zebrafish linkage groups. A c. We reconstructed the HSmap from its original set of markers and combined the HSmap markers with the new, RAD-seq markers and built a second map. LG20 shows very close agreement between markers in both maps, as indicated by red lines that connect identical markers in the two maps.

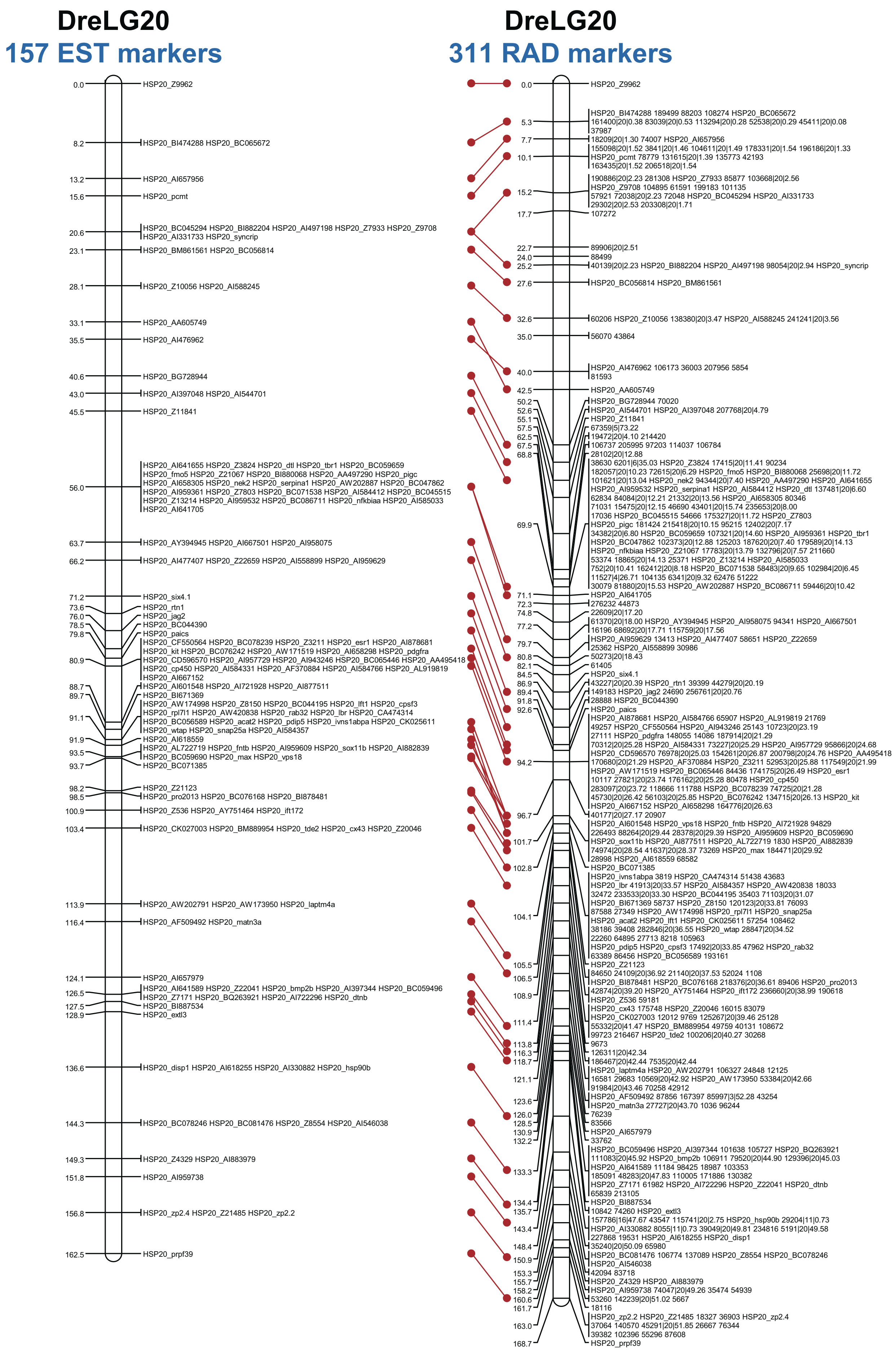
DreLG20
157 EST markers
DreLG20
311 RAD markers
0.0 HSP20_Z9962
8.2 HSP20_BI474288 HSP20_BC065672
13.2 HSP20_AI657956
15.6 HSP20_pcmt
20.6 HSP20_BC045294 HSP20_BI882204 HSP20_AI497198 HSP20_Z7933 HSP20_Z9708 HSP20_AI331733 HSP20_syncrip
23.1 HSP20_BM861561 HSP20_BC056814
28.1 HSP20_Z10056 HSP20_AI588245
33.1 HSP20_AA605749
35.5 HSP20_AI476962
40.6 HSP20_BG728944
43.0 HSP20_AI397048 HSP20_AI544701
45.5 HSP20_Z11841
56.0 HSP20_AI641655 HSP20_Z3824 HSP20_dtl HSP20_tbr1 HSP20_BC059659 HSP20_fmo5 HSP20_Z21067 HSP20_BI880068 HSP20_AA497290 HSP20_pigc HSP20_AI658305 HSP20_nek2 HSP20_serpina1 HSP20_AW202887 HSP20_BC047862 HSP20_AI959361 HSP20_Z7803 HSP20_BC071538 HSP20_AI584412 HSP20_BC045515 HSP20_Z13214 HSP20_AI959532 HSP20_BC086711 HSP20_nfkbiaa HSP20_AI585033 HSP20_AI641705
63.7 HSP20_AY394945 HSP20_AI667501 HSP20_AI958075
66.2 HSP20_AI477407 HSP20_Z22659 HSP20_AI558899 HSP20_AI959629
71.2 HSP20_six4.1
73.6 HSP20_rtn1
76.0 HSP20_jag2
78.5 HSP20_BC044390
79.8 HSP20_paics
80.9 HSP20_CF550564 HSP20_BC078239 HSP20_Z3211 HSP20_esr1 HSP20_AI878681 HSP20_kit HSP20_BC076242 HSP20_AW171519 HSP20_AI658298 HSP20_pdgfra HSP20_CD596570 HSP20_AI957729 HSP20_AI943246 HSP20_BC065446 HSP20_AA495418 HSP20_cp450 HSP20_AI584331 HSP20_AF370884 HSP20_AI584766 HSP20_AL919819 HSP20_AI667152
88.7 HSP20_AI601548 HSP20_AI721928 HSP20_AI877511
89.7 HSP20_BI671369
91.1 HSP20_AW174998 HSP20_Z8150 HSP20_BC044195 HSP20_lft1 HSP20_cpsf3 HSP20_rpl7l1 HSP20_AW420838 HSP20_rab32 HSP20_lbr HSP20_CA474314 HSP20_BC056589 HSP20_acat2 HSP20_pdip5 HSP20_ivns1abpa HSP20_CK025611 HSP20_wtap HSP20_snap25a HSP20_AI584357
91.9 HSP20_AI618559
93.5 HSP20_AL722719 HSP20_fntb HSP20_AI959609 HSP20_sox11b HSP20_AI882839 HSP20_BC059690 HSP20_max HSP20_vps18
93.7 HSP20_BC071385
98.2 HSP20_Z21123
98.5 HSP20_pro2013 HSP20_BC076168 HSP20_BI878481
100.9 HSP20_Z536 HSP20_AY751464 HSP20_ift172
103.4 HSP20_CK027003 HSP20_BM889954 HSP20_tde2 HSP20_cx43 HSP20_Z20046
113.9 HSP20_AW202791 HSP20_AW173950 HSP20_laptm4a
116.4 HSP20_AF509492 HSP20_matn3a
124.1 HSP20_AI657979
126.5 HSP20_AI641589 HSP20_Z22041 HSP20_bmp2b HSP20_AI397344 HSP20_BC059496 HSP20_Z7171 HSP20_BQ263921 HSP20_AI722296 HSP20_dtnb
127.5 HSP20_BI887534
128.9 HSP20_extl3
136.6 HSP20_disp1 HSP20_AI618255 HSP20_AI330882 HSP20_hsp90b
144.3 HSP20_BC078246 HSP20_BC081476 HSP20_Z8554 HSP20_AI546038
149.3 HSP20_Z4329 HSP20_AI883979
151.8 HSP20_AI959738
156.8 HSP20_zp2.4 HSP20_Z21485 HSP20_zp2.2
162.5 HSP20_prpf39

Figure S3 HSmap versus RADmap. We reconstructed the HSmap from its original set of markers and combined the HSmap markers with the new, RAD-seq markers and built a second map. LG20, shown here, and all other linkage groups, shows very close agreement between markers in both maps, as indicated by red lines that connect identical markers in the two maps.
