# Supplementary material for: Stacks: Building and Genotyping Loci De Novo From Short-Read Sequences
Source: G3 (Bethesda). 2011 Aug 1;1(3):171–82. doi: 10.1534/g3.111.000240 (PMC3276136; doi:10.1534/g3.111.000240)
Supplement: Supporting Information [file supp_1.3.171_FigureS1.pdf]

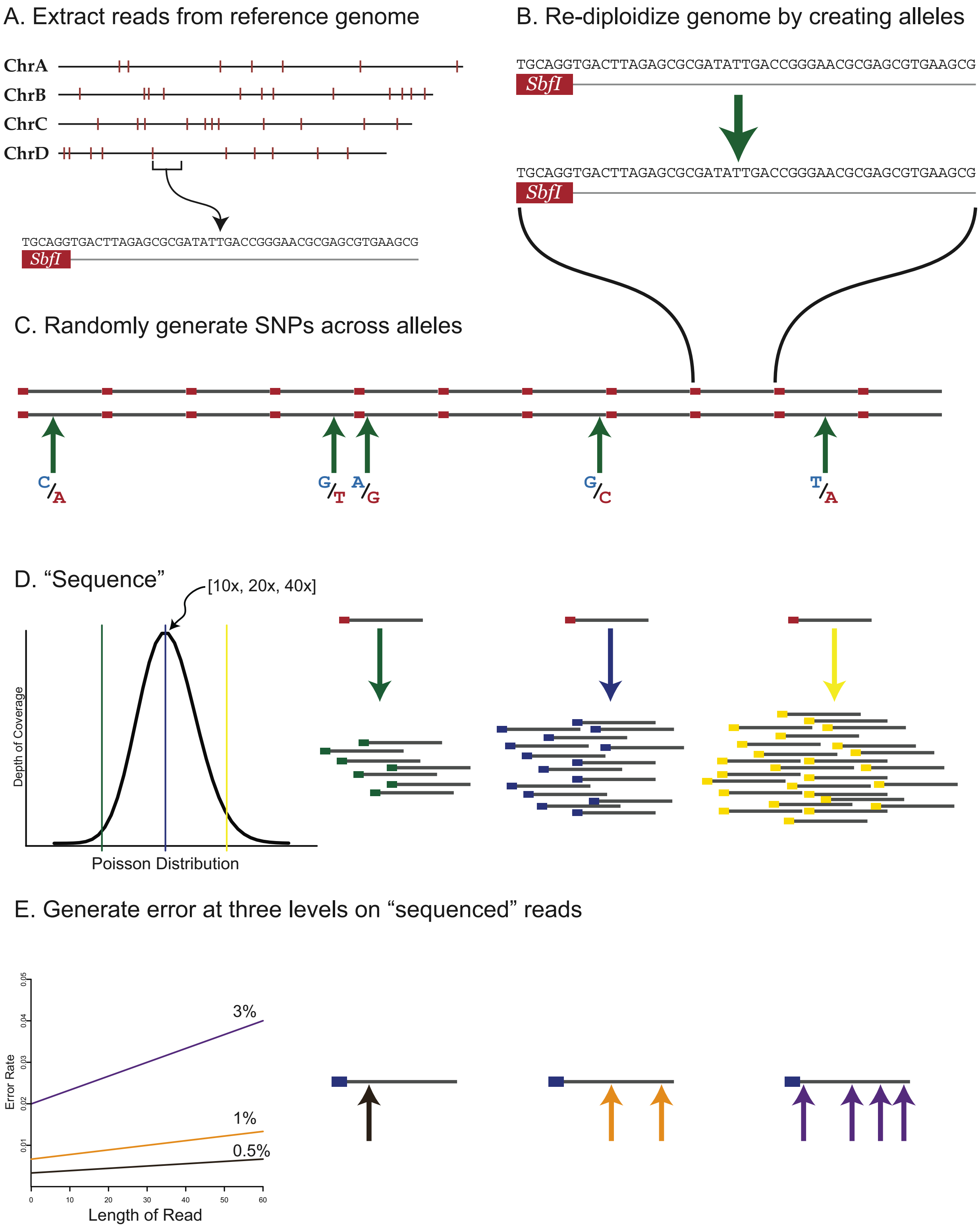

Figure S1 RAD-seq Simulation. (A) 60bp reads were extracted *in silico* from the stickleback reference genome at each occurrence of an *SbfI* restriction enzyme cut site. (B) Extracted reads were re-diplodized and (C) SNPs were added to the reads at a uniform rate of 0.5%. (D) The reads were "sequenced" at per-allele mean depths of 10x, 20x, and 40x by drawing numbers from a Poisson distribution. (E) Errors were added to the "sequenced" reads at three rates, 0.5%, 1%, and 3%.
