# Supplementary material for: Stacks: Building and Genotyping Loci De Novo From Short-Read Sequences
Source: G3 (Bethesda). 2011 Aug 1;1(3):171–82. doi: 10.1534/g3.111.000240 (PMC3276136; doi:10.1534/g3.111.000240)
Supplement: Supporting Information [file supp_1.3.171_FigureS2.pdf]

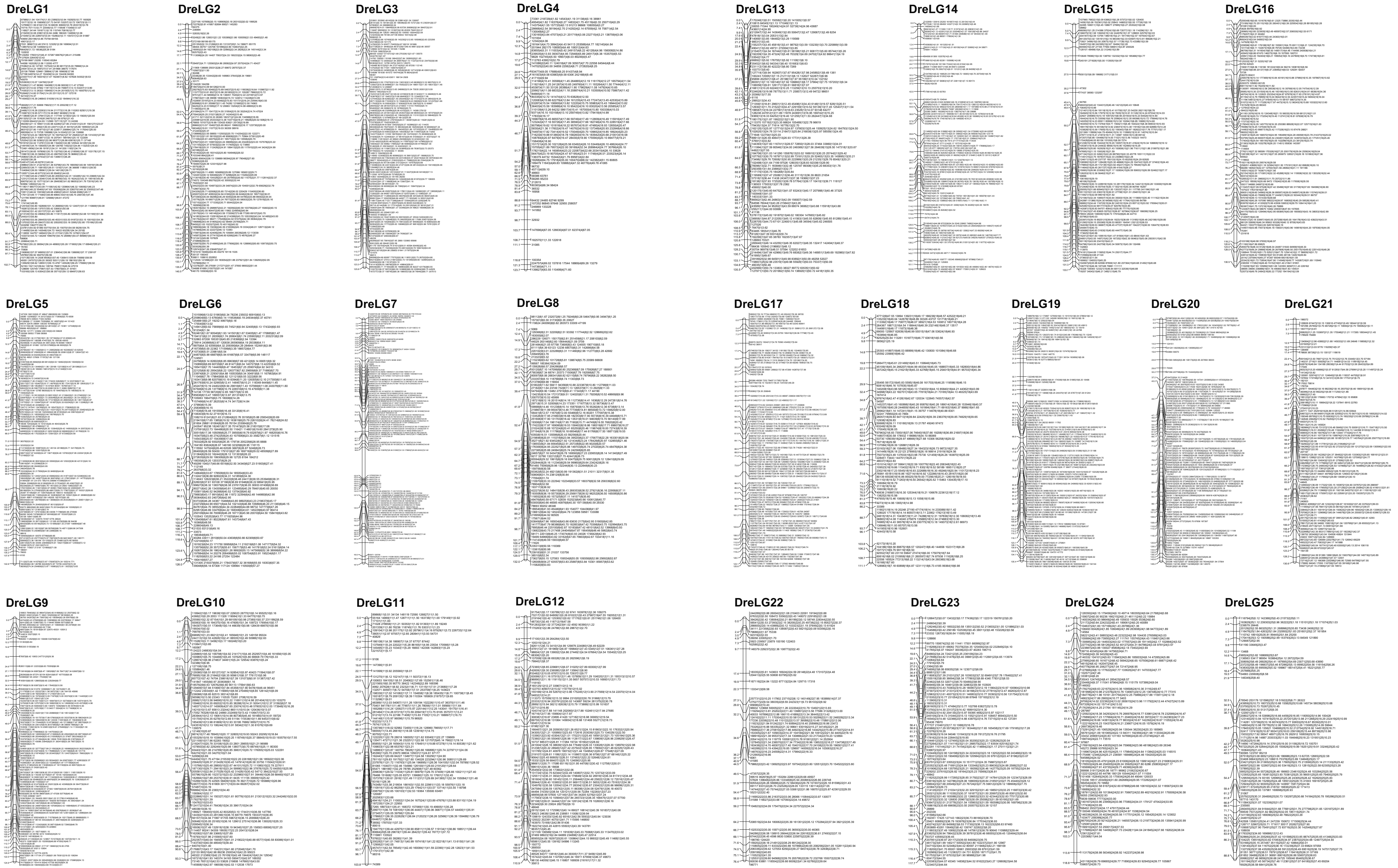

Figure S2 *Danio rerio* RADmap. We mapped 7,861 markers generated from a doubled haploid mapping panel; analysis by *Stacks* recapitulated the 25 zebrafish linkage groups. A c. We reconstructed the HSmap from its original set of markers and combined the HSmap markers with the new, RAD-seq markers and built a second map. LG20 shows very close agreement between markers in both maps, as indicated by red lines that connect identical markers in the two maps.
