# Supplementary material for: Stacks: Building and Genotyping Loci De Novo From Short-Read Sequences
Source: G3 (Bethesda). 2011 Aug 1;1(3):171–82. doi: 10.1534/g3.111.000240 (PMC3276136; doi:10.1534/g3.111.000240)
Supplement: Supporting Information [file supp_1.3.171_FigureS3.pdf]

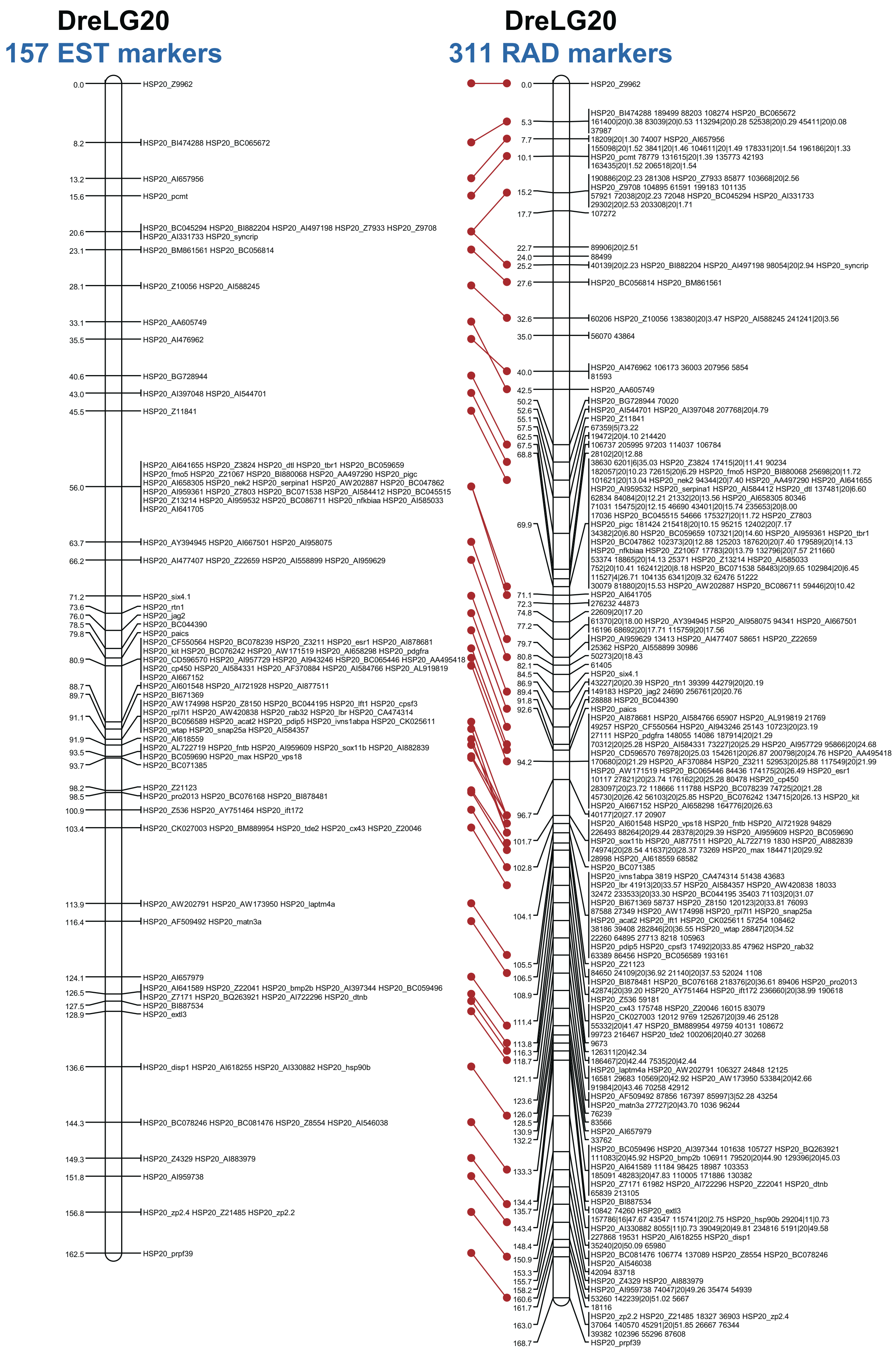

DreLG20
157 EST markers
0.0 HSP20_Z9962
8.2 HSP20_BI474288 HSP20_BC065672
13.2 HSP20_AI657956
15.6 HSP20_pcmt
20.6 HSP20_BC045294 HSP20_BI882204 HSP20_AI497198 HSP20_Z7933 HSP20_Z9708
HSP20_AI331733 HSP20_syncrip
23.1 HSP20_BM861561 HSP20_BC056814
28.1 HSP20_Z10056 HSP20_AI588245
33.1 HSP20_AA605749
35.5 HSP20_AI476962
40.6 HSP20_BG728944
43.0 HSP20_AI397048 HSP20_AI544701
45.5 HSP20_Z11841
56.0 HSP20_AI641655 HSP20_Z3824 HSP20_dtl HSP20_tbr1 HSP20_BC059659
HSP20_fmo5 HSP20_Z21067 HSP20_BI880068 HSP20_AA497290 HSP20_pigc
HSP20_AI658305 HSP20_nek2 HSP20_serpina1 HSP20_AW202887 HSP20_BC047862
HSP20_AI959361 HSP20_Z7803 HSP20_BC071538 HSP20_AI584412 HSP20_BC045515
HSP20_Z13214 HSP20_AI959532 HSP20_BC086711 HSP20_nfkbiaa HSP20_AI585033
HSP20_AI641705
63.7 HSP20_AY394945 HSP20_AI667501 HSP20_AI958075
66.2 HSP20_AI477407 HSP20_Z22659 HSP20_AI558899 HSP20_AI959629
71.2 HSP20_six4.1
73.6 HSP20_rtn1
76.0 HSP20_jag2
78.5 HSP20_BC044390
79.8 HSP20_paics
80.9 HSP20_CF550564 HSP20_BC078239 HSP20_Z3211 HSP20_esr1 HSP20_AI878681
HSP20_kit HSP20_BC076242 HSP20_AW171519 HSP20_AI658298 HSP20_pdgfra
HSP20_CD596570 HSP20_AI957729 HSP20_AI943246 HSP20_BC065446 HSP20_AA495418
HSP20_cp450 HSP20_AI584331 HSP20_AF370884 HSP20_AI584766 HSP20_AL919819
HSP20_AI667152
88.7 HSP20_AI601548 HSP20_AI721928 HSP20_AI877511
89.7 HSP20_BI671369
91.1 HSP20_AW174998 HSP20_Z8150 HSP20_BC044195 HSP20_lft1 HSP20_cpsf3
HSP20_rpl7l1 HSP20_AW420838 HSP20_rab32 HSP20_lbr HSP20_CA474314
HSP20_BC056589 HSP20_acat2 HSP20_pdip5 HSP20_ivns1abpa HSP20_CK025611
HSP20_wtap HSP20_snap25a HSP20_AI584357
91.9 HSP20_AI618559
93.5 HSP20_AL722719 HSP20_fntb HSP20_AI959609 HSP20_sox11b HSP20_AI882839
HSP20_BC059690 HSP20_max HSP20_vps18
93.7 HSP20_BC071385
98.2 HSP20_Z21123
98.5 HSP20_pro2013 HSP20_BC076168 HSP20_BI878481
100.9 HSP20_Z536 HSP20_AY751464 HSP20_ift172
103.4 HSP20_CK027003 HSP20_BM889954 HSP20_tde2 HSP20_cx43 HSP20_Z20046
113.9 HSP20_AW202791 HSP20_AW173950 HSP20_laptm4a
116.4 HSP20_AF509492 HSP20_matn3a
124.1 HSP20_AI657979
126.5 HSP20_AI641589 HSP20_Z22041 HSP20_bmp2b HSP20_AI397344 HSP20_BC059496
HSP20_Z7171 HSP20_BQ263921 HSP20_AI722296 HSP20_dtnb
127.5 HSP20_BI887534
128.9 HSP20_extl3
136.6 HSP20_disp1 HSP20_AI618255 HSP20_AI330882 HSP20_hsp90b
144.3 HSP20_BC078246 HSP20_BC081476 HSP20_Z8554 HSP20_AI546038
149.3 HSP20_Z4329 HSP20_AI883979
151.8 HSP20_AI959738
156.8 HSP20_zp2.4 HSP20_Z21485 HSP20_zp2.2
162.5 HSP20_prpf39
DreLG20
311 RAD markers
0.0 HSP20_Z9962
5.3 HSP20_BI474288 189499 88203 108274 HSP20_BC065672
161400|20|0.38 83039|20|0.53 113294|20|0.28 52538|20|0.29 45411|20|0.08
37987
7.7 18209|20|1.30 74007 HSP20_AI657956
10.1 155098|20|1.52 3841|20|1.46 104611|20|1.49 178331|20|1.54 196186|20|1.33
HSP20_pcmt 78779 131615|20|1.39 135773 42193
163435|20|1.52 206518|20|1.54
15.2 190886|20|2.23 281308 HSP20_Z7933 85877 103668|20|2.56
HSP20_Z9708 104895 61591 199183 101135
57921 72038|20|2.23 72048 HSP20_BC045294 HSP20_AI331733
29302|20|2.53 203308|20|1.71
17.7 107272
22.7 89906|20|2.51
24.0 88499
25.2 40139|20|2.23 HSP20_BI882204 HSP20_AI497198 98054|20|2.94 HSP20_syncrip
27.6 HSP20_BC056814 HSP20_BM861561
32.6 60206 HSP20_Z10056 138380|20|3.47 HSP20_AI588245 241241|20|3.56
35.0 56070 43864
40.0 HSP20_AI476962 106173 36003 207956 5854
81593
42.5 HSP20_AA605749
50.2 HSP20_BG728944 70020
52.6 HSP20_AI544701 HSP20_AI397048 207768|20|4.79
55.1 HSP20_Z11841
57.5 67359|5|73.22
62.5 19472|20|4.10 214420
67.5 106737 205995 97203 114037 106784
68.8 28102|20|12.88
69.9 38630 6201|6|35.03 HSP20_Z3824 17415|20|11.41 90234
182057|20|10.23 72615|20|6.29 HSP20_fmo5 HSP20_BI880068 25698|20|11.72
101621|20|13.04 HSP20_nek2 94344|20|7.40 HSP20_AA497290 HSP20_AI641655
HSP20_AI959532 HSP20_serpina1 HSP20_AI584412 HSP20_dtl 137481|20|6.60
62834 84084|20|12.21 21332|20|13.56 HSP20_AI658305 80346
71031 15475|20|12.15 46690 43401|20|15.74 235653|20|8.00
17036 HSP20_BC045515 54666 175327|20|11.72 HSP20_Z7803
HSP20_pigc 181424 215418|20|10.15 95215 12402|20|7.17
34382|20|6.80 HSP20_BC059659 107321|20|14.60 HSP20_AI959361 HSP20_tbr1
HSP20_BC047862 102373|20|12.88 125203 187620|20|7.40 179589|20|14.13
HSP20_nfkbiaa HSP20_Z21067 17783|20|13.79 132796|20|7.57 211660
53374 18865|20|14.13 25371 HSP20_Z13214 HSP20_AI585033
752|20|10.41 162412|20|8.18 HSP20_BC071538 58483|20|9.65 102984|20|6.45
11527|4|26.71 104135 6341|20|9.32 62476 51222
30079 81880|20|15.53 HSP20_AW202887 HSP20_BC086711 59446|20|10.42
71.1 HSP20_AI641705
72.3 276232 44873
74.8 22609|20|17.20
77.2 61370|20|18.00 HSP20_AY394945 HSP20_AI958075 94341 HSP20_AI667501
16196 68692|20|17.71 115759|20|17.56
79.7 HSP20_AI959629 13413 HSP20_AI477407 58651 HSP20_Z22659
25362 HSP20_AI558899 30986
80.8 50273|20|18.43
82.1 61405
84.5 HSP20_six4.1
86.9 43227|20|20.39 HSP20_rtn1 39399 44279|20|20.19
89.4 149183 HSP20_jag2 24690 256761|20|20.76
91.8 28888 HSP20_BC044390
92.6 HSP20_paics
94.2 HSP20_AI878681 HSP20_AI584766 65907 HSP20_AL919819 21769
49257 HSP20_CF550564 HSP20_AI943246 25143 10723|20|23.19
27111 HSP20_pdgfra 148055 14086 187914|20|21.29
70312|20|25.28 HSP20_AI584331 73227|20|25.29 HSP20_AI957729 95866|20|24.68
HSP20_CD596570 76978|20|25.03 154261|20|26.87 200798|20|24.76 HSP20_AA495418
170680|20|21.29 HSP20_AF370884 HSP20_Z3211 52953|20|25.88 117549|20|21.99
HSP20_AW171519 HSP20_BC065446 84436 174175|20|26.49 HSP20_esr1
10117 27821|20|23.74 176162|20|25.28 80478 HSP20_cp450
283097|20|23.72 118666 111788 HSP20_BC078239 74725|20|21.28
45730|20|26.42 56103|20|25.85 HSP20_BC076242 134715|20|26.13 HSP20_kit
HSP20_AI667152 HSP20_AI658298 164776|20|26.63
96.7 40177|20|27.17 20907
101.7 HSP20_AI601548 HSP20_vps18 HSP20_fntb HSP20_AI721928 94829
226493 88264|20|29.44 28378|20|29.39 HSP20_AI959609 HSP20_BC059690
HSP20_sox11b HSP20_AI877511 HSP20_AL722719 1830 HSP20_AI882839
74974|20|28.54 41637|20|28.37 73269 HSP20_max 184471|20|29.92
28998 HSP20_AI618559 68582
102.8 HSP20_BC071385
104.1 HSP20_ivns1abpa 3819 HSP20_CA474314 51438 43683
HSP20_lbr 41913|20|33.57 HSP20_AI584357 HSP20_AW420838 18033
32472 233533|20|33.30 HSP20_BC044195 35403 71103|20|31.07
HSP20_BI671369 58737 HSP20_Z8150 120123|20|33.81 76093
87588 27349 HSP20_AW174998 HSP20_rpl7l1 HSP20_snap25a
HSP20_acat2 HSP20_lft1 HSP20_CK025611 57254 108462
38186 39408 282846|20|36.55 HSP20_wtap 28847|20|34.52
22260 64895 27713 8218 105963
HSP20_pdip5 HSP20_cpsf3 17492|20|33.85 47962 HSP20_rab32
63389 86456 HSP20_BC056589 193161
105.5 HSP20_Z21123
106.5 84650 24109|20|36.92 21140|20|37.53 52024 1108
HSP20_BI878481 HSP20_BC076168 218376|20|36.61 89406 HSP20_pro2013
108.9 42874|20|39.20 HSP20_AY751464 HSP20_ift172 236660|20|38.99 190618
HSP20_Z536 59181
111.4 HSP20_cx43 175748 HSP20_Z20046 16015 83079
HSP20_CK027003 12012 9769 125267|20|39.46 25128
55332|20|41.47 HSP20_BM889954 49759 40131 108672
99723 216467 HSP20_tde2 100206|20|40.27 30268
113.8 9673
116.3 126311|20|42.34
118.7 186467|20|42.44 7535|20|42.44
121.1 HSP20_laptm4a HSP20_AW202791 106327 24848 12125
16581 29683 10569|20|42.92 HSP20_AW173950 53384|20|42.66
91984|20|43.46 70258 42912
123.6 HSP20_AF509492 87856 167397 85997|3|52.28 43254
HSP20_matn3a 27727|20|43.70 1036 96244
126.0 76239
128.5 83566
130.9 HSP20_AI657979
132.2 33762
133.3 HSP20_BC059496 HSP20_AI397344 101638 105727 HSP20_BQ263921
111083|20|45.92 HSP20_bmp2b 106911 79520|20|44.90 129396|20|45.03
HSP20_AI641589 11184 98425 18987 103353
185091 48283|20|47.83 110005 171886 130382
HSP20_Z7171 61982 HSP20_AI722296 HSP20_Z22041 HSP20_dtnb
65839 213105
134.4 HSP20_BI887534
135.7 10842 74260 HSP20_extl3
143.4 157786|16|47.67 43547 115741|20|2.75 HSP20_hsp90b 29204|11|0.73
HSP20_AI330882 8055|11|0.73 39049|20|49.81 234816 5191|20|49.58
227868 19531 HSP20_AI618255 HSP20_disp1
148.4 35240|20|50.09 65980
150.9 HSP20_BC081476 106774 137089 HSP20_Z8554 HSP20_BC078246
HSP20_AI546038
153.3 42094 83718
155.7 HSP20_Z4329 HSP20_AI883979
158.2 HSP20_AI959738 74047|20|49.26 35474 54939
160.6 53260 142239|20|51.02 5667
161.7 18116
163.0 HSP20_zp2.2 HSP20_Z21485 18327 36903 HSP20_zp2.4
37064 140570 45291|20|51.85 26667 76344
39382 102396 55296 87608
168.7 HSP20_prpf39

Figure S3 HSmap versus RADmap. We reconstructed the HSmap from its original set of markers and combined the HSmap markers with the new, RAD-seq markers and built a second map. LG20, shown here, and all other linkage groups, shows very close agreement between markers in both maps, as indicated by red lines that connect identical markers in the two maps.
